# Dynamic consolidated bioprocessing for innovative lab-scale production of bacterial alkaline phosphatase from *Bacillus paralicheniformis* strain APSO

**DOI:** 10.1038/s41598-021-85207-4

**Published:** 2021-03-16

**Authors:** Soad A. Abdelgalil, Nadia A. Soliman, Gaber A. Abo-Zaid, Yasser R. Abdel-Fattah

**Affiliations:** 1 Bioprocess Development Department, Genetic Engineering and Biotechnology Research Institute (GEBRI), City for Scientific Research and Technological Applications, Alexandria, Egypt; 2 Present Address: New Borg El-Arab City, Universities and Research Institutes Zone, PostAlexandria, 21934 Egypt

**Keywords:** Biochemistry, Biotechnology, Microbiology, Pollution remediation

## Abstract

To meet the present and forecasted market demand, bacterial alkaline phosphatase (ALP) production must be increased through innovative and efficient production strategies. Using sugarcane molasses and biogenic apatite as low-cost and easily available raw materials, this work demonstrates the scalability of ALP production from a newfound *Bacillus paralicheniformis* strain APSO isolated from a black liquor sample. Mathematical experimental designs including sequential Plackett–Burman followed by rotatable central composite designs were employed to select and optimize the concentrations of the statistically significant media components, which were determined to be molasses, (NH_4_)_2_NO_3_, and KCl. Batch cultivation in a 7-L stirred-tank bioreactor under uncontrolled pH conditions using the optimized medium resulted in a significant increase in both the volumetric and specific productivities of ALP; the alkaline phosphatase throughput 6650.9 U L^−1^, and µ = 0.0943 h^−1^; respectively, were obtained after 8 h that, ameliorated more than 20.96, 70.12 and 94 folds compared to basal media, PBD, and RCCD; respectively. However, neither the increased cell growth nor enhanced productivity of ALP was present under the pH-controlled batch cultivation. Overall, this work presents novel strategies for the statistical optimization and scaling up of bacterial ALP production using biogenic apatite.

## Introduction

The bioeconomy represents the value chain of sustainable manufacturing using renewable, low-cost biological resources to sustainably produce food, energy, and industrial products^1^. Bioeconomic process innovations have included processing technologies that use raw and residual biogenic materials as the starting source and biobased processes that take advantage of living organisms’ metabolic behaviors, such as microorganisms, bacteria, fungi, or algae^2^. The bioeconomy is expected to replace old industries, products, and practices with new eco-friendly industries, bioproducts, and procedures, thus enhancing the sustainability of the production and consumption processes while meeting market feedstock demand and price^3^.

Designing effective and cost-competitive green technologies capable of sustainably producing bioproducts from biomass is a key challenge facing a rising bioeconomy. Green chemistry is an eco-friendly approach that maintains sustainability by efficiently using raw materials, eliminating waste, and avoiding the use and generation of substances toxic or hazardous to human health and the environment in the manufacturing and application of biobased products^4^.

In Egypt, a significant amount of biogenic apatite (i.e., bone) waste is produced from animal slaughterhouses, food manufacturing facilities, and glue facilities, causing environmental pollution. Biogenic apatite is effective and vital phosphorus (P) resource that can be recycled; biogenic apatite has a similar calcium (Ca) and P content as natural phosphate rocks (25–29% Ca and 15–19% P) and has fewer impurities than mined phosphate, and thus requires less intensive beneficiation^5^. Additionally, industrial sugar production from beets and sugarcane generates molasses, an opaque, nutrient-rich liquid, as a byproduct, thus providing a renewable, reliable, readily available, and low-cost raw material that can be used to feed most microorganisms, as it contains carbon, nitrogen, phosphorus, sodium, potassium, and magnesium. A concentrated sugar substrate such as molasses can be used to obtain a high yield of bio-based products via industrial-scale fermentation^6^.

There are now robust and comprehensive methods of biocatalysis for chemical synthesis and transformation, contaminant bioremediation, and sustainable energy production, thus reinforcing and enhancing the potential of green technology for environmental purposes^4^. Enzymatic bioremediation is a vital branch of green chemistry used to clean contaminated sites in which microorganisms enzymatically attack recalcitrant environmental pollutants and break them down or convert them to innocuous products^7^.

One such bioremediation enzyme that can be used to reduce environmental pollution and degradation is alkaline phosphatases (ALP; EC 3.1.3.1.). ALP is an example of orthophosphate monoester phosphohydrolases; it is a metalloenzyme, non-specific, and a phosphomonoesterase that shows optimal activity at an alkaline pH^8^. ALP is a homodimeric enzyme consisting of two similar monomers that contain five cysteine residues, two zinc atoms, and one magnesium atom, which provides it with its catalytic functionality. ALPs catalyze the hydrolysis of the C–O–P linkage in a variety of phosphate esters via a phosphoryl intermediate to produce a free phosphate ion (i.e., dephosphorylation) and a hydrolyzed molecule, or catalyze a transphosphorylation reaction in the presence of large concentrations of phosphate acceptors. ALP has a broad range of substrate specificity and can be used on synthetic as well as natural substrates^9^.

Several prokaryotes from bacteria (e.g., *Escherichia coli*, *Bacillus species*, *Mycobacterium smegmatis*, *Thermotoga maritime*, *Haloarcula marismortui*) to mammals (e.g., humans) produce ALPs, as these stable enzymes can remove phosphate groups from diverse molecules, including nucleotides, proteins, and alkaloids^10^, although they are most predominant in *Escherichia coli* and calf intestine. However, their inherently low thermal stability and shelving lives have limited the implementation of ALPs^8^, as practical implementation requires usage under harsh environments. Thermostable ALPs, especially those from various *Bacillus* species, are thus favored for genetic engineering, bioengineering, and industrial applications. However, few researchers have addressed the extracellular production of ALP from the *Bacillus* genus; the *Bacillus licheniformis’* ALP’s extracellular production is 10 times as high as other species^11^. The scale-up of a bioprocess is critical in determining the economic viability of the bioproduct concerned and is an important step in transferring the bioprocess from the laboratory to the industrial scale^12^ However, scaling up the production of bacterial ALP has not yet been addressed.

**Figure 1:**
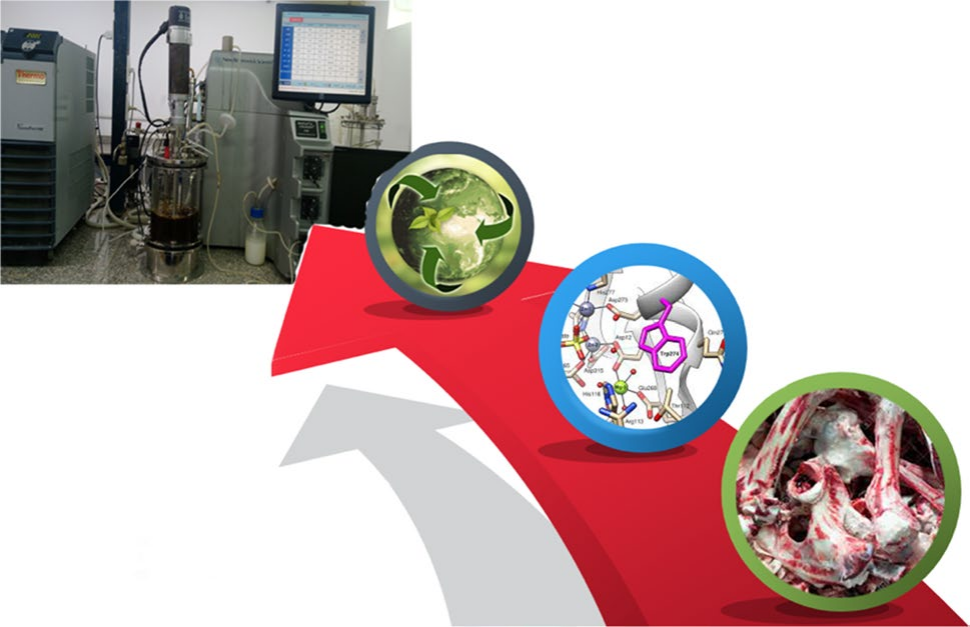
Schematic illustration of biogenic apatite waste utilization to scale up the production of bacterial ALP from the *Bacillus paralicheniformis* strain APSO to the bench-top bioreactor scale for green chemistry sustainability.

This work, therefore, aims to better utilize food industrial waste (biogenic apatite) and agro-industrial waste (sugarcane molasses) to scale up the production of bacterial ALP from the *Bacillus paralicheniformis* strain APSO to the benchtop bioreactor scale by optimizing growth conditions to increase the specific phosphatase activity as illustrated in Fig. 1. Mathematical experimental designs including the Plackett–Burman design (PBD) and response surface methodology (RSM) are employed to optimize the media constituents during the fermentation process to address the shortcomings of the traditional one-factor-at-a-time approach (OFAT) and to enhance metabolite productivity^13^.

## Results and discussion

### Isolation and identification of phosphatase-producing bacteria

**Figure 2:**
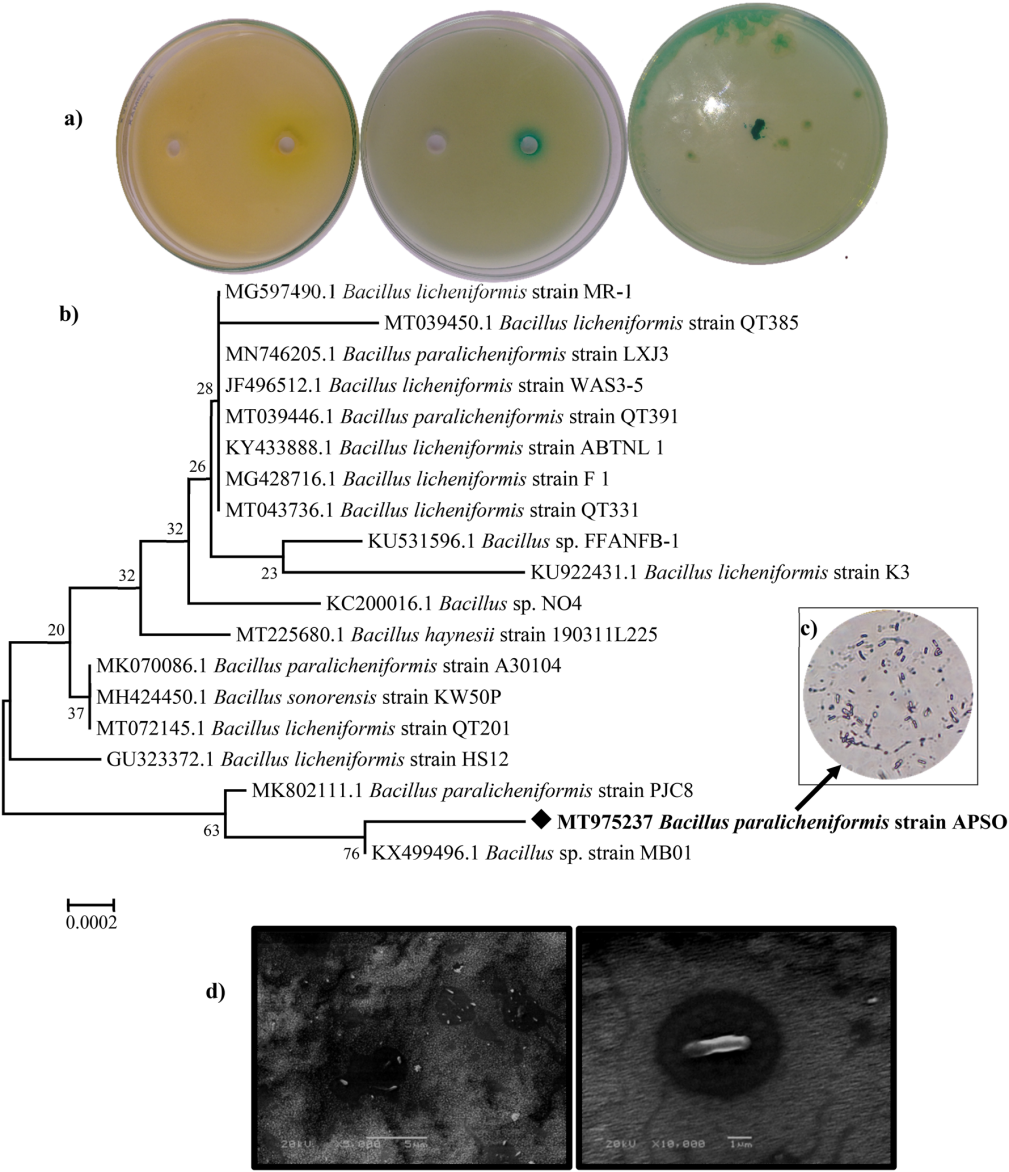
(**a**) Qualitative screening of alkaline phosphatase (ALP)-producing bacteria. (**b**) Phylogenetic tree based on 16 S rDNA gene-sequencing analysis showing the relationship of the Bacillus paralicheniformis strain APSO with reference strains (NCBI GenBank) constructed using the neighbor-joining method with the aid of the MEGA 7.0 program. Sequence divergence is indicated by the scale bar. (**c**) Gram-stain of the PL isolate (100 × magnification via an oil lens). (**d**) Scanning electron microscopy (SEM) 20-kV micrograph showing the morphology of the cell at magnifications of 5000 × and 1000 ×.

Modified Pikovskaya’s (PVK) broth amended with animal bone powder as a phosphate source was used as a medium in a program for exploring the potential production of ALP to enrich and accelerate the growth of the naturally occurring ALP-producing bacteria present in a black liquor sample. Twenty-five bacterial isolates were obtained from the enrichment isolation process in the primary exploration phase. To screen ALP-producing isolates from the obtained isolates, the plate agar media assay used an artificial substrate that yielded a colored product like the yellow color of the para-nitrophenol end product as a result of para-nitrophenyl phosphate (pNPP) cleavage and a deep-green color as a result of cleavage of phenolphthalein diphosphate tetrasodium salt (PDP) in presences of methyl green (MG) as an indicator dye, as detailed by Patel Falguni and others^14^ for selecting phosphatase producers. Thus, the formation of deep-green/yellow-stained colonies indicated a positive production. Among the twenty-five isolates, four showed a particularly high ALP activity and were graded according to the degree of green/yellow staining of colonies. The isolate entitled PL exhibited intense bluish-green colored spots on MG-PDP plates, as shown in Fig. 2a, and showed remarkable potency for ALP productivity (approximately 12.51 U L^–1^ min^–1^ versus the other three isolates’ activities of 8.14, 7.84, and 6.38 U L^–1^ min^–1^. The PL isolate was thus selected for further study. Based on the cultural and morphological characterization, the PL isolate was tentatively assigned to the genus *Bacillus licheniformis;* this assignment was confirmed via Basic Local Alignment Search Tool (BLAST) analysis data of the 16S rRNA gene sequence, which showed 99.72% similarity with the genus *Bacillus paralicheniformis*. Sanger’s dideoxynucleotide sequencing of the amplified 16S rRNA gene resulted in a 1447-bps nucleotide sequence.

The 16S rRNA gene sequences of members of the genus *Bacillus* were retrieved from the NCBI database and used as references for consolidating the phylogenetic position of the strain through a phenogram tree. The PL isolate was closest in homology to *Bacillus paralicheniformis* strain PJC8 with an identity of 99.45% and query cover of 100% as illustrated in Fig. 2b. The 16S rRNA gene sequence was submitted to the GenBank, NCBI, and assigned the accession number MT975237, identifying it as *Bacillus paralicheniformis* strain APSO (MT975237). Figures 2c and d demonstrate the Gram staining micrograph and the shape of strain PL via scanning electron microscopy (SEM) with magnification 10,000 ×, respectively. The identified bacteria were Gram-positive, small, and rod-shaped (0.64 in width × 1.26 μm in length) endospore-forming bacilli with a characterized halo-zone around the cell. ALP has been reported from several *Bacillus* species, including *Bacillus licheniformis*^15^ and *Bacillus subtilis*^16^.

### Effect of physical parameters on ALP productivity

As physical parameters are among the most critical variables in the production of ALPs, the influence of various physical parameters including temperature, pH, and initial inoculum percentage on ALP production by *Bacillus paralicheniformis* strain APSO was investigated. Of the three studied fermentation temperatures (40 °C, 45 °C, and 50 °C), *Bacillus paralicheniformis* strain APSO showed the highest phosphatase activity (25.17 U L^–1^) at 45 °C. At 50 °C, the productivity of ALP decreased fivefold (5.2 U L^–1^), whereas it decreased by a factor of 1.5 (16.37 U L^–1^) at 40 °C. These results are consistent with those obtained by Behera and others^16^, who reported that the maximum yield of ALP from *Alcaligenes faecalis* was achieved at 45 °C. The pH of the fermentation broth influences the metabolic activity of microorganisms and thus plays a significant role in the optimization of the fermentation processes; the catalytic activity of ALP has been reported to be regulated mainly by Tris buffers^18^. The obtained results demonstrated that the maximum ALP productivity was recorded at pH values of 9 and 10 (31.7 and 27.19 U L^–1^, respectively), with a notable falling off in productivity of ALP for slightly acidic (pH 6.0), neutral (pH 7.0) and slightly alkaline (pH 8.0) conditions to activities of 6.64, 11.46, and 15.76 U L^–1^, respectively. *Bacillus paralicheniformis* APSO thus is confirmed to have a strong preference for alkaline conditions for ALP production; this is in accordance with other published reports detailing the necessity of alkaline conditions (pH 8.0 and 9.0) to obtain the maximum production of ALP by *Bacillus subtilis*^16^*, Bacillus licheniformis*^15^, and *Bacillus flexus*^14^. Of the activated pre-cultured inoculum amounts studied (1%, 2%, 5%, and 10% of the total fermentation broth), the highest ALP productivities (70.4 and 61.84 U L^–1^ min^–1^) were recorded when 5% and 10% of the fermentation broth was activated pre-cultured inoculum, respectively. At 1% and 2% inoculum sizes, the production of enzyme substantially decreased to 4.0 and 4.5 U L^–1^, respectively. These results are in good agreement with those obtained by Priya et al.^19^ and Jatoth et al.^16^, who found that an inoculum size of 5% optimized ALP productivity by *Bacillus megaterium* and *Bacillus subtilis*, respectively.

### Statistical optimization of ALP production by *Bacillus paralicheniformis* strain APSO

Maintaining optimum and homogenous reaction conditions of the fermentation process minimizes the chance of microbial stress exposure, enhances metabolic accuracy, and ensures consistent yield and product quality, and is thus considered the main goal of scaling up fermentation processes.

In the present investigation, the significance of eight different nutrimental parameters (namely; molasses, (NH_4_)_2_NO_3_, NaCl, MgSO_4_.7H_2_O, animal bone powder, KCl, CoCl_2_.6H_2_O, and MnSO_4_.H_2_O, corresponding to *X*_*1*_–*X*_*8*_, respectively) on the production of alkaline phosphatase was screened to improve the composition of the medium by simultaneous comparisons between two levels (high; + 1 and low; − 1 values) of above-nominated factors by applying Plackett–Burman design to the fifteen experimental trials. The layout of experimental PBD for the screening of significant variables along with their corresponding ALP throughput and residuals are shown in Table 1. A wide variation in alkaline phosphatase productivity all over the different experiments ranging from 11 to 2468.8 U L^-1^ was noticed from the obtained results in Table 1, which highlighted the importance of further media optimization to attain a high yield of the interested product. The difference between the two levels of each independent variable (+ 1 and − 1) was chosen to be large enough to ensure that it includes the peak area for the maximum enzyme production.

**Table 1:** Randomized PBD for evaluating factors influencing ALP production by *Bacillus paralicheniformis* strain APSO.

Trails	Variables								ALP activity (U L^−1^ min^−1^)		
	X1	X2	X3	X4	X5	X6	X7	X8	Actual value	Predicted value	Residual
1	0	0	0	0	0	0	0	0	886.11	818.70	67.40
2	1	− 1	− 1	− 1	1	− 1	− 1	1	904.44	820.79	83.65
3	− 1	1	− 1	1	1	1	− 1	− 1	194.94	415.66	− 220.72
4	1	1	1	− 1	− 1	− 1	1	− 1	1618.5	1839.28	− 220.72
5	1	− 1	− 1	1	− 1	1	1	1	1154.9	1542.30	− 387.30
6	1	− 1	1	1	1	− 1	− 1	− 1	957	1055.20	− 98.20
7	1	1	− 1	− 1	− 1	1	− 1	− 1	2117.5	1911.33	206.16
8	− 1	1	1	1	− 1	− 1	− 1	1	8.84	91.78	− 82.93
9	− 1	1	− 1	− 1	1	− 1	1	1	11	109.20	− 98.20
10	1	1	1	1	1	1	1	1	2468.8	2096.13	372.75
11	0	0	0	0	0	0	0	0	800.55	818.70	− 18.15
12	− 1	− 1	1	− 1	1	1	1	− 1	119.47	202.40	− 82.93
13	− 1	− 1	1	− 1	− 1	1	− 1	1	18.94	− 49.43	68.379
14	0	0	0	0	0	0	0	0	856.77	818.70	38.07
15	− 1	− 1	− 1	1	− 1	− 1	1	− 1	162.55	− 210.20	372.75

Variable	Code	Coded and actual levels									
		− 1	0	1							
Molasses	X1	5	15	25							
(NH_4_)_2_NO_3_	X2	0.1	0.3	0.5							
NaCl	X3	0.1	0.3	0.5							
MgSO_4_.7H_2_O	X4	0.02	0.11	0.2							
Animal bone powder	X5	0.1	1.02	2							
KCl	X6	0.1	0.3	0.5							
CoCl_2_.6H_2_O	X7	0.0005	0.0015	0.0025							
MnSO_4_.H_2_O	X8	0.0005	0.0015	0.0025							

**Figure 3:**
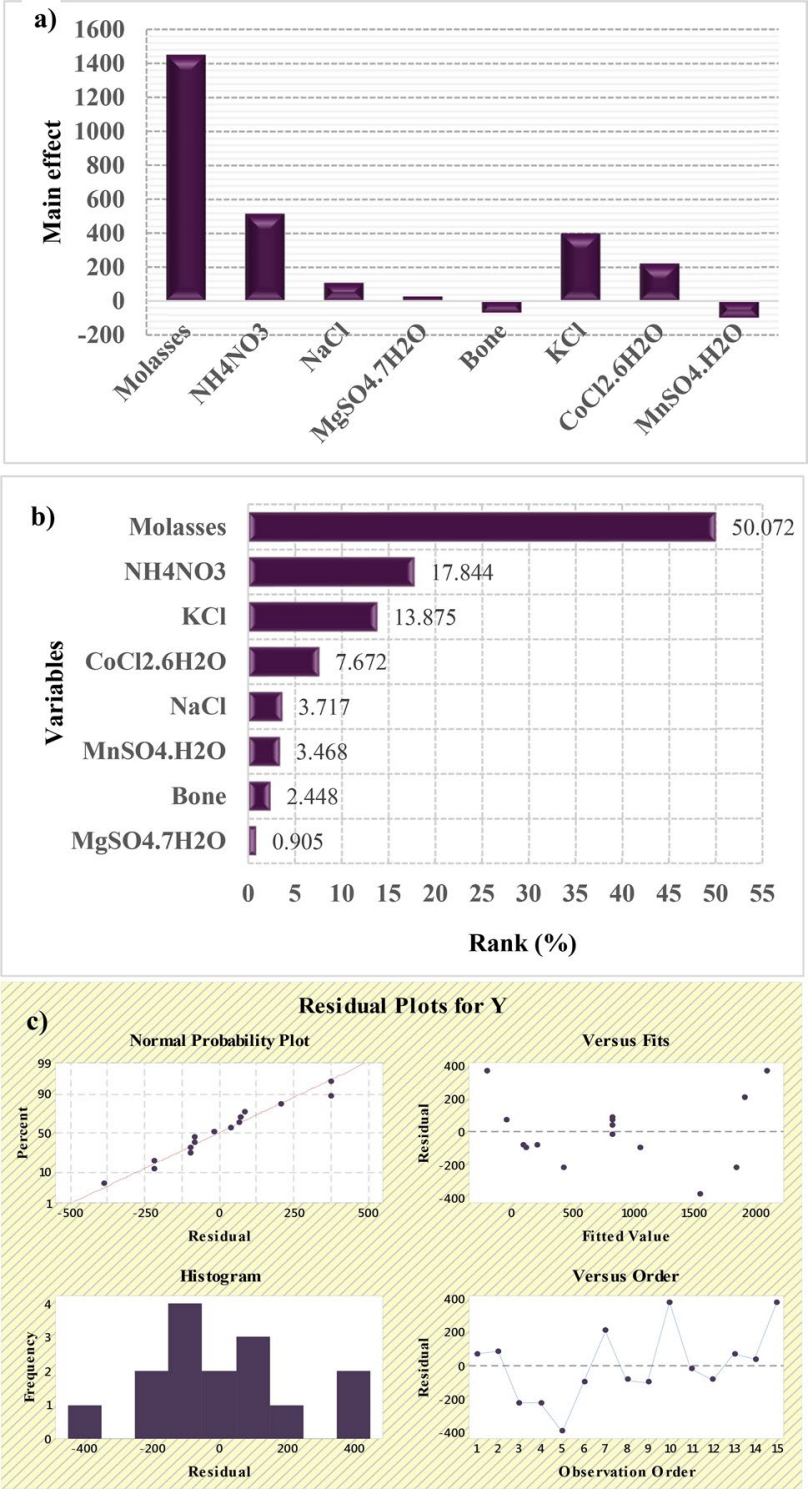
Results obtained from the Plackett–Burman design (PBD): (**a**) main culture variables, (**b**) Pareto chart illustrating the order and significance of the variables affecting laccase production by *Bacillus paralicheniformis* strain APSO in a ranking percentage from 0.905 to 50.072, and (**c**) normal probability plot of the residuals for laccase production determined by the first-order polynomial equation.

The concentration of molasses had a clear impact on ALP activity; when a high concentration amount of molasses was used (e.g., trials 10 and 4), a maximum activity of 2468.8 U L^–1^ occurred, whereas using less molasses (e.g., trial 9) led to lower activity, 11 U L^–1^. Molasses thus plays an indispensable role in stimulating ALP production by *Bacillus paralicheniformis* strain APSO. The adequacy of the model was calculated, the variables evidencing statistically significant effects were screened via *t*-test, and analysis of variance (ANOVA) was performed; the resulting coefficients and *t*- and *p*-values are shown in Table 2. Here, the *p*-value represents the probability of error of the respective variable in the population and is thus used to evaluate the significance of each of the coefficients. The *t*-test for any individual effect allows an evaluation of the probability of finding the observed effect purely by chance. The parameters characterized by *p*-value < 0.05 with confidence levels greater than 95% and a high value of *t*-test were considered to significantly influencing the activity. The main effect of the variables under investigation was estimated by ∑(+ 1)/n _(+1)_ – ∑(–1)/n _(–1)_; the results are summarized graphically in Fig. 3a. Based on the ANOVA, molasses (*X*_*1*_), (NH_4_)_2_NO_3_ (*X*_*2*_), and KCl (*X*_*6*_) were determined to significantly contribute to the production process (*p*-values of 0.00022, 0.0316, and 0.0732, respectively, with a contribution ratio of 99.977%, 96.835%, and 92.676%, respectively). However, CoCl_2_.6H_2_O, NaCl, and MgSO_4_.7H_2_O were also determined to be moderately significant, with contribution ratios of 72.42%, 41.75%, and 10.78%, respectively, whereas MnSO_4_.H_2_O and animal bone powder were effective at a lower level.

**Table 2:** Statistical analysis of PBD showing coefficients and t- and p-values for each variable affecting ALP production.

Variables	Coefficient	Main effect	Std error	t-Stat	P-value	Confidence level (%)
Intercept	818.7071		82.9247	9.8728	0.0000623	99.993
X1	725.4693	1450.939	92.7127	7.8249	0.0002299	99.977
X2	258.5276	517.0554	92.7127	2.7884	0.031640	96.835
X3	53.8563	107.7128	92.7127	0.5808	0.58245	41.754
X4	13.10915	26.21831	92.7127	0.1413	0.89218	10.781
X5	− 35.4719	− 70.9438	92.7127	− 0.3826	0.71520	28.479
X6	201.0278	402.0557	92.7127	2.1682	0.07323	92.676
X7	111.1498	222.2998	92.7127	1.1988	0.27577	72.422
X8	− 50.2426	− 100.485	92.7127	− 0.5419	0.60739	39.260

ANOVA	df	SS	MS	F	Significance F	
Regression	8	7,833,164	979,145	9.49264	0.0066325	
Residual	6	618,886	103,147			
Total	14	8,452,051				
R Square	0.93					
Adjusted R Square	0.829

A Pareto chart clarifying the influence of the parameters studied on the production process is shown in Fig. 3b, where the bars with a length proportional to the absolute value of the estimated effects divided by the standard error were utilized to construct the chart. The bars are ranked in order of the size of the effects, with the largest effects on the top^20^. The resulting Fisher’s *F*-test value and *p-*value (9.49264 and 0.00663) confirms the significance of the model; there is only a 0.66% chance that a model F-value this large could occur due to noise, and the *p*-value < 0.05. The linear equation representing the production of ALP in terms of the independent variables studied was thus derived, as shown in Eq. (1). As the resulting determination coefficient R^2^ = 93%, this was determined to be a good fit, as only approximately 7.0% of the total variations created by variables do not fit the predicted ALP activity (Table 2).

1 $$Y_{activity} = {818.707} + {725.46}X_{1} + {258.52}X_{2} + {53.85}X_{3} + {13.109}X_{4} - {35.471}X_{5} + {201.02}X_{6} + {111.14}X_{7} - {50.242}X_{8} .$$

To demonstrate the normality of the obtained data set, the distribution residuals are plotted against the expected normal values of the model in Fig. 3c; as the residual points follow the diagonal line indicating the expected ALP productivity, the expected results fit well with the experimental results.

The results of the PBD optimization of the medium were then verified by comparing the productivity of the ALP of the pre-and post-optimized conditions; using the optimized conditions (pH 9.0, 45 °C, inoculum size 5%, v/v, agitation speed 200 rpm, 48 h of incubation time, and medium composition of, in g L^−1^: molasses, 25.0; (NH_4_)_2_NO_3_, 0.5; NaCl, 0.5; MgSO_4_·7H_2_O, 0.11; animal bone powder, 0.1; KCl, 0.5; CoCl_2_.6H_2_O, 0.0025; MnSO_4_.H_2_O, 0.0005) allowed for a 21-fold increase in productivity (from 70.4 to 1475.83 U L^–1^). These results demonstrate the first such optimization of ALP production employing animal bone powder as a biogenic apatite source.

Furthermore, prior efforts to optimize bacterial ALP production have relied on the one factor at once (OFAT) method; this work thus also details the first successful optimization of nutrient ratios for ALP production employing statistical experimental PBD, except for the work by Pandey et al.^15^, who reported the optimization of physical parameters for enhancing ALP production by *Bacillus licheniformis*. Comparing the obtained results with those cited by the other investigators, it was found that the highest level of the *Escherichia coli* Efrl13 alkaline phosphatase production was achieved by using the mineral medium with molasses 20 g L^−1^, and enzyme activity was fallbeyond that concentration as a result of suppression action of molasses^21^. However, Pandey and others^8^ found that using a synthetic rich medium containing, 23.9, 13.5, and 1.5 g L^−1^ glucose, peptone, and yeast extract as sources of carbon and nitrogen, respectively, led to the improved production of ALP from *Bacillus licheniformis* (2670 U mL^−1^), this result is not in conformity with the obtained results of the present study which depended only on biogenic waste residues.

### Response surface methodology via rotatable central composite design

RSM is a mathematical empirical model that uses experimental data to assess the regression model and improve the response (i.e., output) of a variable influenced by several independent input variables^22^. A 2^5–1^ half-fractional factorial rotatable central composite design (RCCD) was employed to fit the multiple regression models of the fermentation study. Following the initial screening via PBD, central-composite design, a commonly used analytical method in RSM, was used to determine the optimal levels of the three significant parameters. As detailed above, molasses (*X*_*1*_), (NH_4_)_2_NO_3_ (*X*_*2*_), and KCl (*X*_*6*_) were the most significant nutrimental variables and were thus selected for further optimization using RCCD. The remaining variables were held constant at the level determined to provide maximum production of ALP as obtained in Plackett–Burman experiments.

The RCCD had six axial, eight factorial, and four center points, resulting in eighteen experimental trials to optimize the chosen variables; each was performed twice. The experimental error was determined by the four replicates at the center points. The design matrix, including the eighteen experimental trials with varying combinations of *X*_*1*_, *X*_*2*_, and *X*_*6*_, and the experimental and predicted ALP production and residuals are summarized in Table 3. Overall, the activity of ALP ranged from 1138 to 3684 U L^–1^; the highest ALP activity (approximately 3675–3684 U L^–1^) was achieved in the center-point trials (trials 6, 7, 10, and 13) at molasses, (NH_4_)_2_NO_3_, and KCl concentrations of 30, 0.9, and 1.2 g L^–1^, respectively, whereas the lowest productivity (1138 U L^–1^) was seen at molasses, (NH_4_)_2_NO_3_, and KCl concentrations of 20, 1.2, and 0.8 g L^–1^, respectively (i.e., trial 2).

**Table 3:** Matrix designed for *Bacillus paralicheniformis* strain APSO RCCD.

Trials	Type	Variables				ALP productivity (U L^−1^ min^−1^)		
		X1		X2	X3	Actual value	Predicted value	Residual
1	Factorial	1-		1-	1-	2019.72	2007.32	12.39993
2	Factorial	− 1		1	− 1	1138.19	1298.23	− 160.044
3	Axial	0		− 1.6817	0	3089.16	2773.44	315.7235
4	Axial	0		1.6817	0	1303.19	1494.83	− 191.642
5	Factorial	1		− 1	1	2209.165	2136.86	72.30523
6	Center	0		0	0	3684.125	3686.72	− 2.59477
7	Center	0		0	0	3675.395	3686.72	− 11.3248
8	Axial	0		0	− 1.6817	2077.775	1757.99	319.7834
9	Axial	1.6817		0	0	1960.135	1711.59	248.5435
10	Center	0		0	0	3682.38	3686.72	− 4.33977
11	Axial	− 1.6817		0	0	1543.055	1667.51	− 124.462
12	Factorial	1		1	1	1225.275	1325.41	− 100.139
13	Center	0		0	0	3683.69	3686.72	− 3.02977
14	Factorial	1		− 1	− 1	1595	2125.19	− 530.195
15	Axial	0		0	1.6817	1650	1845.70	− 195.702
16	Factorial	− 1		− 1	1	2155.69	2312.32	− 156.63
17	Factorial	− 1		1	1	1833.33	1390.87	442.4555
18	Factorial	1		1	− 1	1595	1526.10	68.89131

Variables	Code	Coded and actual levels						
		− 1.6817	− 1	0	1	1.6817		
Molasses	X1	10	20	30	40	50		
(NH_4_)_2_NO_3_	X2	0.3	0.6	0.9	1.2	1.5		
KCl	X3	0.4	0.8	1.2	1.6	2		

### Multiple regression analysis and analysis of variance

The results of the RCCD were then subjected to multiple regression statistical analysis and ANOVA calculations; the results are detailed in Table 4. The model reliability and precision were evaluated via statistical regression analysis parameters, including the determination coefficient (R^2^) value, predicted R^2^ value, adj R^2^ value, F-value, and lack of fit. The most correlation relationship between the experimental findings and the theoretical values predicted of the chosen model was weighted by the highest value for the determination coefficient (R^2^)^23^. As R^2^ = 0.94, the model thus can accurately predict the relationship between the factors influencing the production of ALP, as approximately 94% of the variance in the ALP productivity could be explained by the independent variables studied. Furthermore, the high value of Fisher’s F-test (13.055) and adj R^2^ value (0.864) with, low *p*-value (0.0007), and insignificant lack of fit for ALP productivity (F-value = 2.71; *p*-value = 0.178) support the significance and accuracy of the developed model. The significance of each variable on ALP production (i.e., the *p-*value) are also listed in Table 4; the linear coefficient of (NH_4_)_2_NO_3_ (*X*_*2*_) and the quadratic effects of molasses (*X*_*1*_ × *X*_*1*_), (NH_4_)_2_NO_3_ (*X*_*2*_ × *X*_*2*_), and KCl (*X*_*3*_ × *X*_*3*_) had significant effects on ALP productivity (with corresponding F- and *p*-values of 17.51, 55.96, 33.81 and 49.84, respectively, and 0.0030, 0.00007, 0.000398 and 0.000106, respectively). On the contrary, the effect of mutual interaction between (NH_4_)_2_NO_3_ and KCl (*X*_*2*_ × *X*_*3*_) had F- and *p*-values of 0.2 and 0.666, respectively, and thus did not significantly contribute to the response. The linear coefficients of molasses (*X*_*1*_) and KCl (*X*_*3*_) and the coefficients of interaction between molasses and (NH_4_)_2_NO_3_ (*X*_*1*_ × *X*_*2*_) were characterized by a positive *t*-test value (0.144, 0.287, and 0.231, respectively), indicating their significant contribution to increasing the efficacy of ALP productivity. To predict the overall response within experimental constraints, a second-order polynomial function was fitted to the experimental results, yielding:

2 $$Y \, = \, 7373.44 \, + \, 26.206X_{1} - \, 760.264X_{2} + \, 52.152X_{3} + \, 55X_{1} X_{2} - \, 146.66X_{1} X_{3} - \, 106.1805X_{2} X_{3} - \, 1412.208X_{1}^{2} - \, 1097.84X_{2}^{2} - \, 1332.8064 \, X_{3}^{2} ,$$

where *Y* represents the predicted ALP activity and *X*_*1*_, *X*_*2*_, and *X*_*3*_ represent the coded levels of the molasses, (NH_4_)_2_NO_3_, and KCl, respectively.

**Table 4:** Statistical analysis of the RCCD showing coefficient values and t- and p- values for each variable on ALP activity.

Term	Estimate	Std error	t ratio	Prob >|t|	F ratio	Confidence level (%)
Intercept	3686.72	167.605	21.99648	1.9264E-08		100
X1	13.10348	90.84058	0.144247	0.88887	0.0208	11.11241
X2	− 380.133	90.84058	− 4.18462	0.003060	17.51	99.69397
X3	26.07637	90.84058	0.287056	0.78136	0.082	21.86364
X1*X2	27.5	118.6889	0.231698	0.82258	0.0537	17.74106
X1*X3	− 73.3337	118.6889	− 0.61787	0.553844	0.3818	44.61555
X2*X3	− 53.09	118.6889	− 0.4473	0.666517	0.2001	33.34824
X1*X1	− 706.105	94.38963	− 7.48074	0.00007055	55.96	99.99294
X2*X2	− 548.921	94.38963	− 5.81548	0.0003980	33.81	99.96019
X3*X3	− 666.403	94.38963	− 7.06013	0.000106	49.84	99.98939

	Df	SS	MS	F		Significance F
Regression	9	13,241,601	1,471,289	13.055		0.0007
Residual	8	901,572.1	112,696.5			
Total	17	14,143,173				
R square	0.94					
Adjusted R^2^	0.864					

### Model adequacy checking

**Figure 4:**
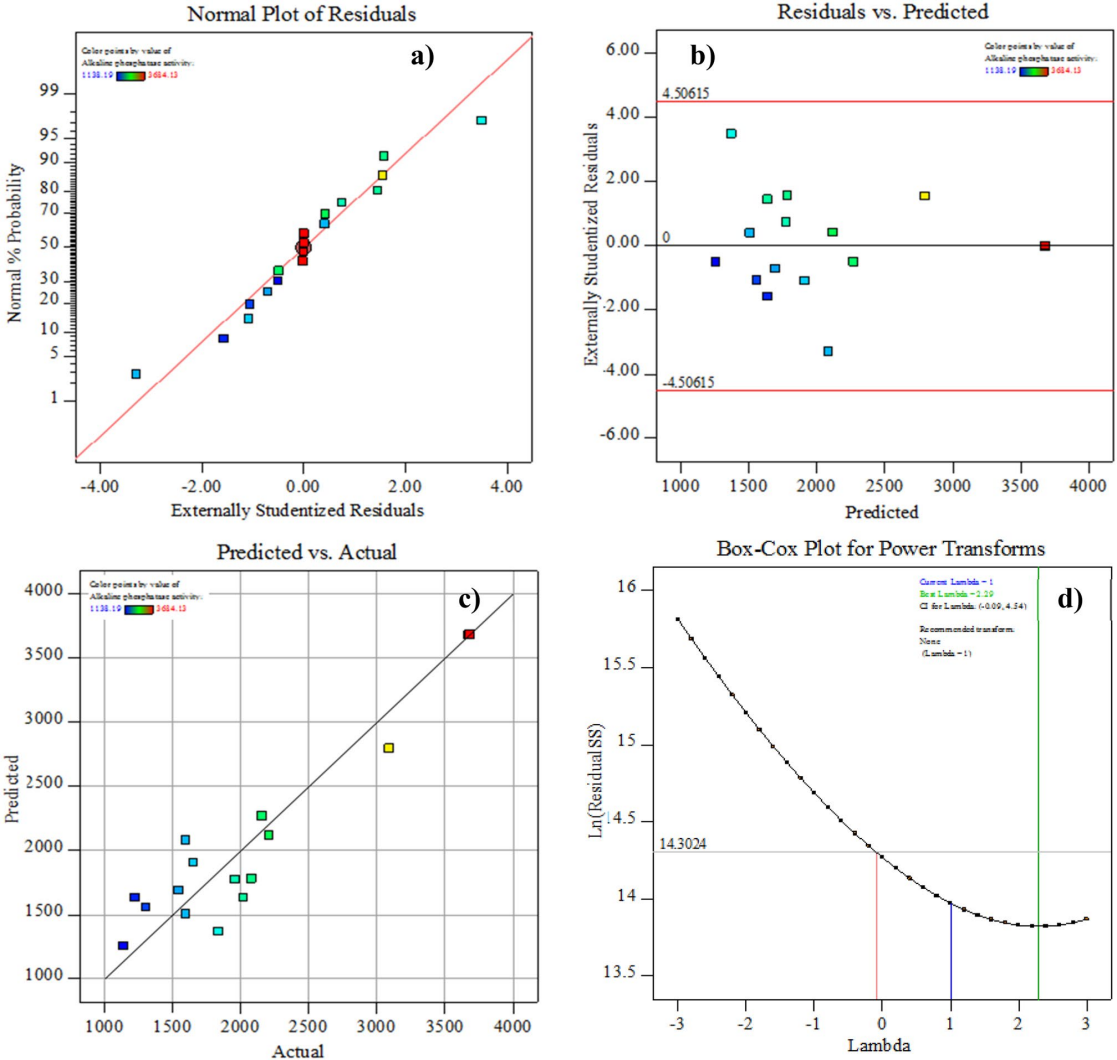
Model adequacy checking of the rotatable central composite design (RCCD): (**a**) normal probability plot of internally studentized residuals, (**b**) internally studentized residuals versus predicted ALP production, (**c**) plot of predicted versus actual ALP production, and **d)** Box-Cox plot of model transformation.

The normal probability chart, shown in Fig. 4a, demonstrates the model appropriateness via the distribution of the residuals along the diagonal line of ALP activity. When plotted against the fitted values of ALP, as shown in Fig. 4b, the residuals fall randomly around the centerline, indicating that they have a steady variance and that the model is adequate and meets the analysis assumptions. Plotting the obtained ALP activity response against the predicted response confirmed the model’s adequacy and the strong consensus between the experimental and theoretical results predicted by the model, as shown in Fig. 4c. From the Box–Cox plot (Fig. 4d), the best lambda (*λ*) value was equal to 2.29, which lies between the points of low and high confidence intervals of – 0.09 of 4.54, respectively, so the data transformation was not recommended.

### Contour and three-dimensional (3D) plots

**Figure 5:**
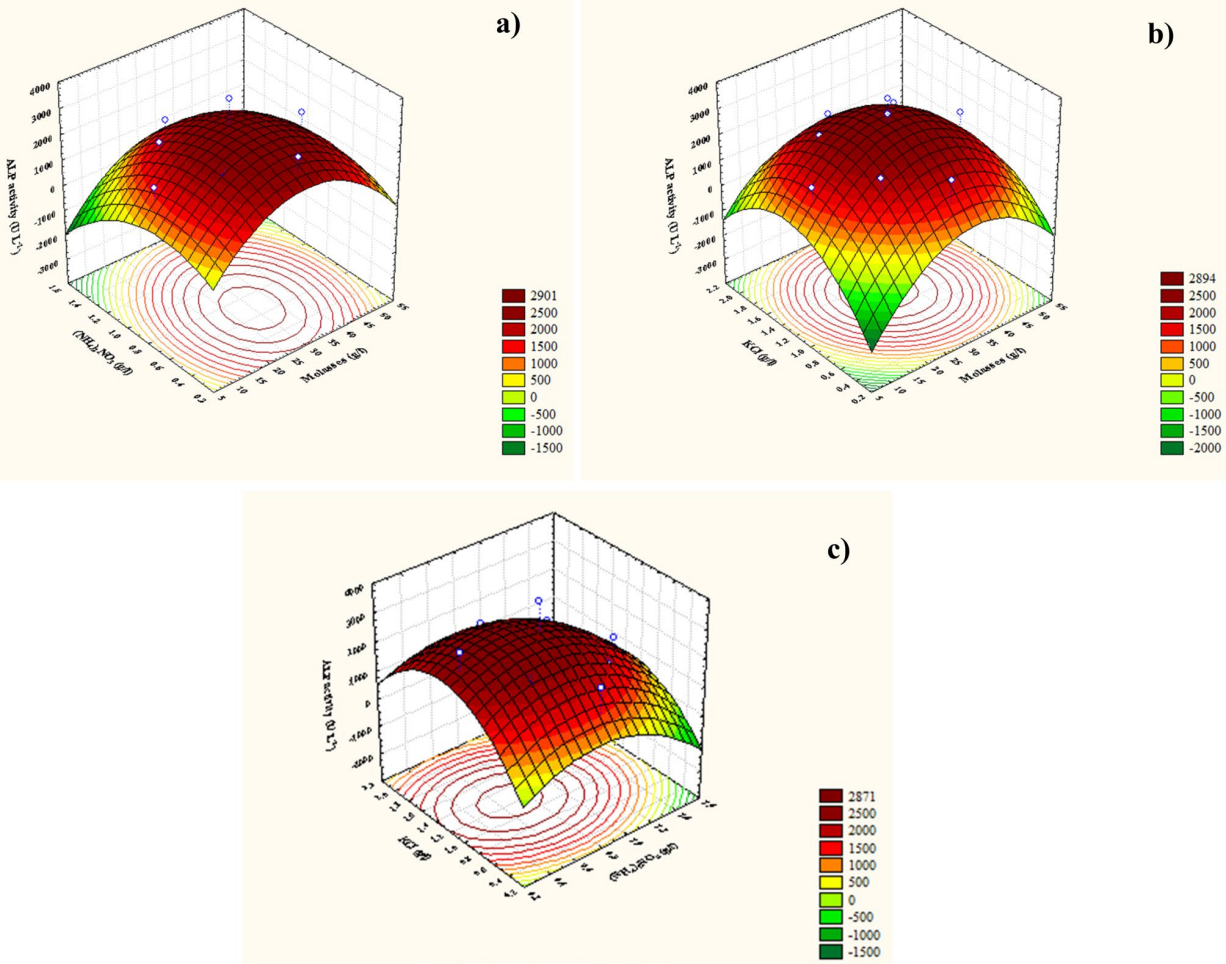
Three-dimensional response surface representing the ALP activity yield (U L^–1^ min^–1^) from *Bacillus paralicheniformis* strain APSO as affected by culture conditions. (**a**) represented the interaction between molasses and (NH_4_)_2_NO_3_, (**b**) represented the interaction between molasses and KCl, and (**c**) represented the interaction between KCl and (NH_4_)_2_NO_3_.

The 3D response surface and 2D graphical contour for the pairwise combinations of the three variables (i.e., *X*_*1*_*X*_*2*_, *X*_*1*_*X*_*3*_, and *X*_*2*_*X*_*3*_) were drawn to obtain optimal conditions for maximum ALP production by identifying the optimal concentrations of each variable under investigation and the effects of their interaction on the expected ALP productivity; results are shown in Fig. 5a–c, respectively. Here, the 3D response plots were constructed by plotting ALP activity on the *z*-axis against the two process variables studied on the *x*- and *y*-axes while keeping the third factor at its zero levels. Thus, in Fig. 5a, KCl was held at its zero level (1.2 g L^−1^) to clarify the effect of molasses and (NH_4_)_2_NO_3_ on ALP production by *Bacillus paralicheniformis* strain APSO. The highest and lowest levels of molasses (*X*_*1*_) were accompanied by a reduction in ALP production, whereas the center point (30 g L^−1^) showed the highest yield of ALP. The highest yield of ALP was recorded at a middle/low concentration of (NH_4_)_2_NO_3_ (0.79 g L^−1^); further increases decreased the ALP productivity. Through point prediction, a maximum predicted ALP activity of 3752.532 U L^–1^ was attained at molasses and (NH_4_)_2_NO_3_ concentrations of 30 and 0.795 g L^−1^, respectively, at the fixed KCl concentration of 1.2 g L^−1^. When the (NH_4_)_2_NO_3_ concentration was held at its zero level (0.9 g L^−1^), a maximum ALP throughput was attained at middle concentrations of molasses (*X*_*1*_) and KCl (*X*_*3*_) (30 and 1.2 g L^−1^, respectively), as shown in Fig. 5b; any further increase or decrease was accompanied by a reduction in the productivity. By solving the Eq. (2) and analyzing Fig. 5b, the maximum predicted ALP productivity of 3686.85 U L^–1^ has been accomplished by using 0.9 g of (NH_4_)_2_NO_3_ at an optimum predicted concentration (g L^−1^) of molasses (30), KCl (1.2). When the molasses concentration (*X*_*1*_) was maintained at its zero level (30 g L^−1^), increasing the KCl concentration increased the ALP productivity at low concentrations of (NH_4_)_2_NO_3_, but increasing the (NH_4_)_2_NO_3_ concentrations above 0.79 g L^−1^ prompted a decline in ALP productivity, as shown in Fig. 5c. The impact of (NH_4_)_2_NO_3_ concentration on ALP productivity was not significant at high KCl concentrations. The highest predicted ALP productivity (3753.274 U L^–1^) thus occurred at KCl and (NH_4_)_2_NO_3_ concentrations of 1.2 and 0.79 g L^−1^, respectively. Moreover, the significance of the correlation between these variables contributes significantly to enhance the productivity of ALP.

### Experimental validation

Validation was performed using a medium composed of, in g L^−1^: molasses, 30; (NH_4_)_2_NO_3_, 0.79; NaCl, 0.5; MgSO_4_.7H_2_O, 0.11; animal bone powder, 0.1; KCl, 1.21; CoCl_2_.6H_2_O, 0.0025; and MnSO_4_.H_2_O, 0.0005. The pH was adjusted to 9.0 by dissolving the media ingredients in 0.1 M Tris-NaOH buffer. 5% of old-activate pre-culture inoculum was used for inoculation and the inoculated medium was incubated at 45 °C and 200 rpm for 24 h.

To validate the developed model, ALP production under the model-predicted optimal conditions (i.e., molasses, (NH_4_)_2_NO_3_, and KCl concentrations of 30, 0.79, and 1.2 g L^−1^) was then experimentally performed. At these conditions, experimental ALP activity of 4042 U L^–1^ was obtained, which is closed to that predicted by the regression model (3753.27 U L^−1^) with 107.69% accuracy, thus confirming the model’s validity. The proposed statistical methodology employing a combination of PBD and RCCD can thus be used to determine the statistically significant factors and find the optimal concentration of these factors. This work thus represents the first use of PBD to improve the productivity of ALP and provides further evidence of the ability of RSM via RCCD to enhance the production process of ALP.

In their efforts to optimize the productivity of ALP from *Bacillus licheniformis*, Pandey et al.^15^ reported a maximum predicted production of ALP of 792.043 U mL^−1^ under pH 8.0, 36.7 °C, a fermentation time of 78 h, and an orbital speed of 165 rpm. Further, when Pandey et al.^8^, employed a CCD to optimize the amount of glucose, peptone, and yeast extract for enhancing the productivity of ALP from *Bacillus licheniformis* output, the determined *p*-values of the coefficient for the linear and interactive effect of glucose, peptone, and yeast extract were all < 0.05; the correlation coefficient (R^2^) was 0.932 (93.2%). Predicted phosphatase activity of 2670.67 U mL^−1^ was obtained at predicted concentrations of glucose, peptone, and yeast extract of 2.39%, 1.35%, and 0.15%, respectively. The methodology presented here, therefore, provides the first demonstrated basis to reduce the production cost of ALP by using agro-industrial waste like molasses and food wastes and optimizing the ratio of nutrient inputs via statistical optimization approaches.

### Scaling up fermentation strategies for ALP production from *Bacillus paralicheniformis* strain APSO

#### Kinetics of cell growth and ALP production in the shake flask under batch conditions

**Figure 6:**
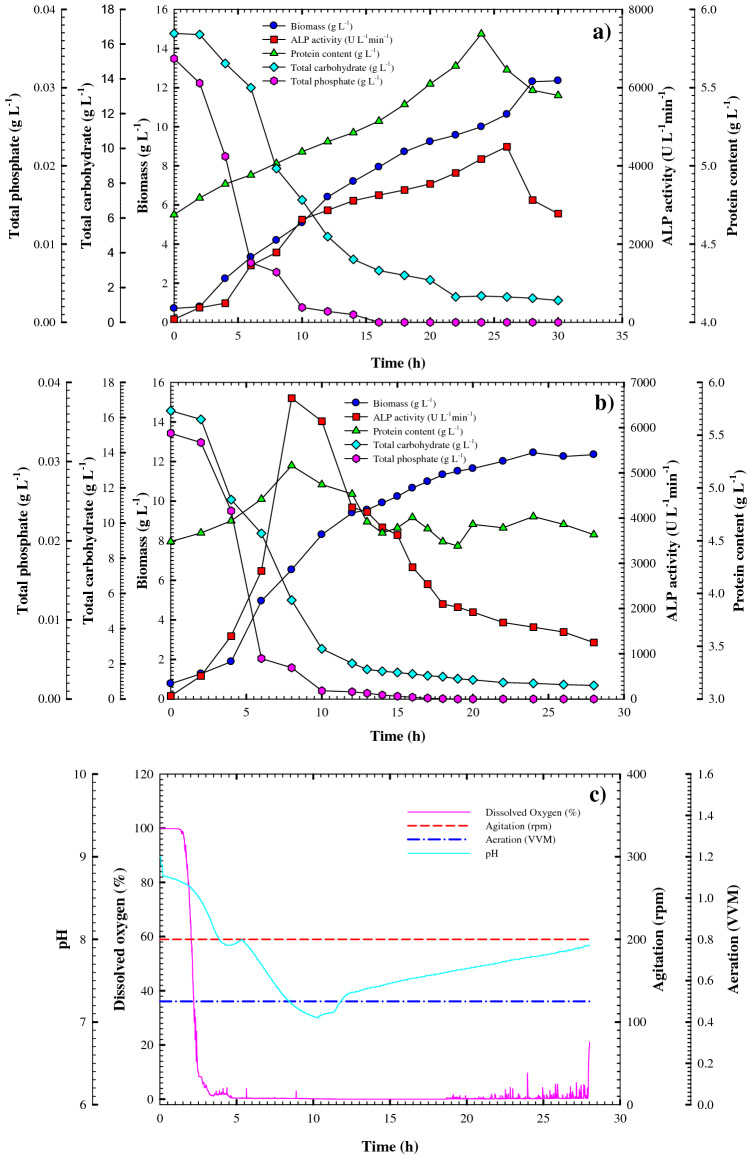
Monitoring of *Bacillus paralicheniformis* strain APSO growth and ALP productivity (**a**) at shake-flask scale cultivation and (**b**) in a 7-L stirred tank bioreactor under uncontrolled pH conditions. (**c**) On-line data (dissolved oxygen, agitation, airflow rate, and pH) as a function of time during batch fermentation in the bioreactor under uncontrolled pH conditions.

During closed batch fermentation, the cell density of the microbial community increases continuously until the limiting nutrients deplete from the medium; this decrease of nutrients is accompanied by an increase in the production of primary and secondary metabolites^24^. Here, small-scale batch cultivation was first performed in a shake-flask to monitor the initial fermentation parameters and enhance the ALP productivity, followed by 7-L benchtop bioreactor batch cultivation for scaling up ALP throughput under uncontrolled and controlled pH submerged fermentation modes. The resulting estimated cell growth kinetics and ALP production are summarized in Table 5, whereas the growth pattern and volumetric and specific ALP production during batch cultivation of *Bacillus paralicheniformis* strain APSO in the optimized media at the shake-flask size at standard cultivation conditions are shown in Fig. 6a. The bacterial cells followed a typical growth pattern that was directly proportional to the ALP productivity. The bacterial cells grew exponentially over time without a significant phase lag at a growth rate of 0.4198 (g L^–1^ h^–1^) and a constant specific growth rate (*µ*) of 0.071 h^–1^. A maximum cell mass of approximately 12.3 g/L was reached after 28 h of cultivation with a yield coefficient *Y*_*x/s*_ = 0.73 (g of cells/g of the substrate), after which point the cell mass remained nearly constant for the rest of the cultivation period. The volumetric ALP production (*Q*_*p*_) reached a peak of 181.78 U L^–1^ h^–1^ after 26 h, corresponding to a maximum production of 4488 U L^–1^ with yield coefficients *Y*_*p/x*_ and *Y*_*p/s*_ of 395.22 and 171.74 U g^–1^, respectively, before gradually decreasing. The protein content reached a maximum value at approximately 26 h of 5.8431 g L^–1^ (from an initial concentration of 4.687 g L^–1^). Meanwhile, a carbohydrate consumption rate of – 0.6290092 g L^–1^ h^–1^ caused a sharp decline in the concentration of total carbohydrates from the initial concentration of 16.607 g L^–1^ to 8.48 g L^–1^ at 8 h of incubation time reaching and 1.25 g L^–1^ at the end of the cultivation period. The total available phosphate also declined rapidly in the first 6 h of cultivation incubation time to 0.0076 g L^–1^; full consumption was reached at 16 h. Throughout the fermentation process, the pH of the culture media gradually decreased from 8.81 to 7.35 at 12 h of cultivation time, as shown in Fig. 6a; this decline in pH was accompanied by a gradual elevation in the bacterial growth and protein content. The pH then gradually increased to 8.22 at 28 h of cultivation time, alongside the complete consumption of available phosphate and the gradual decline in the total carbohydrate concentration, leading to a gradual elevation in ALP productivity. Qureshi et al.^21^ reported that the pH-dependent production of enzymes may be caused by the influence of pH over bacterial growth or pH-dependent control of gene expression for enzyme synthesis. The present finding is analogous to those reported by Butler et al.^25^, who found that phosphatase production was increased during exponential growth of a *Citrobacter* sp.

**Table 5:** Kinetic parameters of cell growth and ALP production by *Bacillus paralicheniformis* strain APSO as affected by different cultivation strategies.

Parameters	Shake Flask Cultivation	Uncontrollable pH Batch Cultivation	Controllable pH Batch Cultivation
**Growth parameters**			
X_max-conc._ (g L^−1^)	12.30196	12.44783	5.06666
µ (h^−1^)	0.071212	0.09433	0.033144
µMax (h^−1^)	0.516556	0.482296	0.402681
dX/dt (g.L^−1^.h^−1^)	0.419790	0.528060	0.122776
**Production parameters**			
P_max_ (IU L^−1^)	4488.266	6650.925	2175.555
P_max.specific_ (IU g^−1^)	799.5154	1276.024	410.93453
P_max.time_ (h)	26	8	20
Q_p_ (IU L^−1^ h^−1^)	181.789	1023.6111	121.7129
− Q_s_ (g.L^−1^.h^−1^)	0.6290092	1.7126057	0.817410
− Q_s_ (g.L^−1^.h^−1^)	0.0012734	0.0047433	0.001257
**Yield coefficient parameters**			
Y_p/s_ (IU g^−1^)	171.74	933.25	98.55
Y_p/x_ (IU g^−1^)	395.22	1042.50	536.1909
Y_x/s_ (g g^−1^)	0.73	0.8	0.23
Overall cultivation time (h)	30	28	28

*X*_max-conc._ maximal cell dry weight, *dx/dt* cell growth rate, *µ* specific growth rate, *P*_*max*_ maximal ALP production, *P*_*max specific*_ specific productivity, *Q*_*p*_ ALP production rate, *Q*_*s*_, substrate consumption rate; *Y*_*p/*x_ U of ALP produced per g biomass, _*p/s*_ U of ALP produced per g substrate consumed, *Y*_*x/s*_, g biomass produced per g substrate consumed.

The metal content obtained via flame atomic adsorption spectrometry at different points throughout the fermentation process is summarized in Table 6. As bacterial ALP contains Zn^2+^ and Mg^2+^^8^, the growth pattern of ALP was synchronous with an increasing concentration of Sn, Zn, Mg, and Mn ions, which reached an apex at 26 h of 283, 22, 652.5, and 4.27 mg L^–1^, respectively, corresponding to the peak ALP production, before decreasing until the end of the fermentation period. This finding is in agreement with Chaudhuri et al.^26^, who documented the necessity of metal ion chelation for enzyme production or stabilization. Meanwhile, Fe ions reached a peak concentration of 20.89 mg L^–1^ at 14 h before decreasing. No changes were observed in the concentrations of Co, Pb, Ni, Cr, Li, or Cd ions throughout the fermentation period, implying that they do not play an essential role in the production of ALP or bacterial growth.

**Table 6:** Flame atomic absorption spectrometry for heavy metal analysis of shake-flask batch cultivation.

Metal ions (mg L^−1^)	Incubation time (h)				
	0	8	14	26	30
Co	< 0.1836	< 0.1836	< 0.1836	< 0.1836	< 0.1836
Cu	< 0.0430	< 0.0430	0.285	< 0.0430	< 0.0430
Pb	< 0.6017	< 0.6017	< 0.6017	< 0.6017	< 0.6017
Fe	9.84	4.312	20.89	19.41	5.99
Ni	< 0.2669	< 0.2669	< 0.2669	< 0.2669	< 0.2669
Sn	148.2	143.27	151.15	283.35	142.2
Cr	< 0.1926	< 0.1926	< 0.1926	< 0.1926	< 0.1926
Zn	3.92	16.93	15.9625	22.05	13.7425
Mg	521.25	495	596.25	652.5	498.75
Mn	0.6575	1.19	1.382	4.27	2.3
Li	< 0.1236	< 0.1236	< 0.1236	< 0.1236	< 0.1236
Cd	< 0.0249	< 0.0247	< 0.0249	< 0.0249	< 0.0249

#### Kinetics of cell growth and ALP production in the bioreactor under uncontrolled pH batch conditions

As the use of the optimized medium greatly enhanced the volumetric productivity and specific production of ALP by *Bacillus paralicheniformis* strain APSO in the above shake-flask batch cultivation, this process was scaled up to a 7-L stirred-tank benchtop bioreactor with a working volume of 4 L under uncontrolled pH for further development and optimization. The resulting relationships between cell growth, enzyme production, and substrate consumption as a function of fermentation time are demonstrated in Fig. 6b. Overall, growth patterns similar to those seen in the shake flask were present; the cells grew exponentially with a growth rate of 0.528 g L^–1^ h^–1^) for a maximum biomass production *X*_*ma*x_ = 12.44 g L^–1^. The obtained yield coefficient *Y*_*x/s*_ = 0.8 (g of cells/g of the substrate) was slightly higher than that of the shake flask, and the specific growth rate (*µ* = 0.094 h^–1^) increased by a factor of 1.32. The peak biomass concentration was reached at 24 h, four hours earlier than in the shake flask.

Meanwhile, ALP production was shifted toward an early exponential phase, demonstrating that bioreactor cultivation resulted in a significant increase and improvement in the volumetric productivity of ALP over shake-flask cultivation. The volumetric productivity of ALP reached a maximum of 6650.9 U L^–1^ at 8 h, which was approximately 48.18% higher and 18 h earlier than in the shake flask. This was accompanied by a considerable elevation in production rate (*Q*_*p*_) and yield coefficients of *Y*_*p/x*_ and *Y*_*p*/s_ to 1023.61 U L^–1^ h^–1^, 1042.5 U g^–1^, and 933.25 U g^–1^, respectively, representing an increase by a factor of 5.63, 2.6, and 5.43, respectively. The total carbohydrate and available phosphate concentrations also showed a marked decrease by 8 h of cultivation to 5.6 and 0.0039 g L^–1^, respectively, from their initial concentrations of 16.3 and 0.03357 g L^–1^, respectively, and reached 0.767 and 0 g L^–1^, respectively by the end of the fermentation period. Overall, the total carbohydrate and phosphate consumption rates were – 1.71 and 0.0047 g L^–1^ h^–1^, respectively, representing an increase by a factor of 2.75 and 3.7, respectively, from the shake-flask cultivation.

The protein content was synchronous with ALP production and thus reached a peak concentration of 5.212 g L^–1^ at 8 h from an initial concentration of 4.491 g L^–1^, causing an increase in the specific productivity of ALP to 1276 U g^–1^. The ALP yield and protein content then declined until the end of the cultivation period. The dissolved oxygen content, shown in Fig. 6c, sharply decreased after 2 h of cultivation from 100 to 10% by 8 h of fermentation time as a result of the high consumption of dissolved oxygen. Afterward, the dissolved oxygen content fluctuated before gradually increasing to 90% by the end of the fermentation period. Fluctuations in the pH were similar to those observed in the shake flask (i.e., a gradual decrease reaching 7.1 at 10 h of cultivation time before a gradual increase to 7.92 at the end of the cultivation). Overall, the increase in maximum volumetric productivity by a factor of 1.48 from that obtained in shake-flask cultivation (6650.9 and 4488.266 U L^–1^, respectively) is attributable to good cultivation of oxygenation and involvement conditions as well as the vessel's bioreactor capacity.

#### Kinetics of cell growth and ALP production in the bioreactor under controlled pH batch conditions

**Figure 7:**
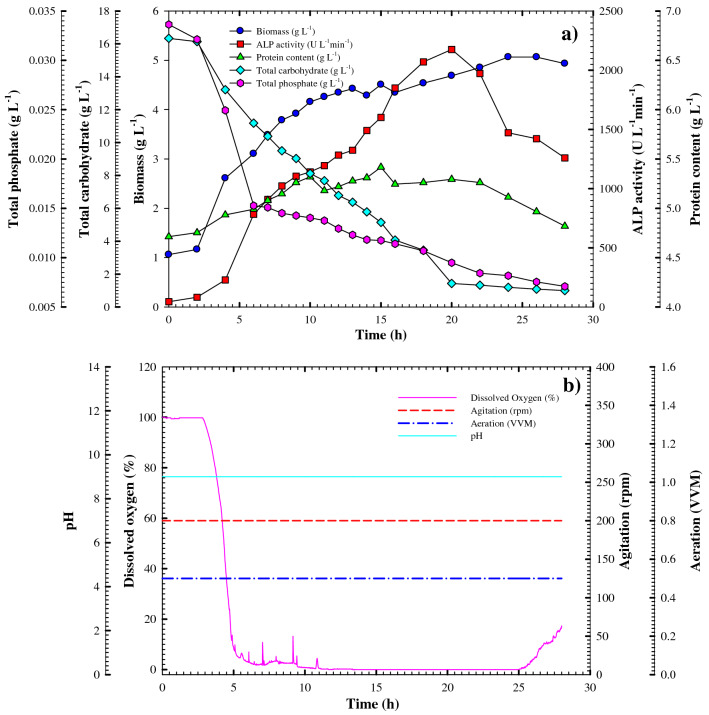
(**a**) Growth and ALP productivity monitoring of *Bacillus paralicheniformis* strain APSO in a 7 L stirred tank bioreactor under a controlled pH. (**b**) On-line data (dissolved oxygen, agitation, airflow rate, and pH) as a function of time during said batch fermentation.

The aforementioned results in bioreactor batch cultivation demonstrated a significant increase in both the volumetric and specific productivities of ALP. To clarify the effect of pH on the cultivation process, the cultivation of *Bacillus paralicheniformis* strain APSO was then performed in the 7-L stirred-tank benchtop bioreactor under controlled pH conditions; the resulting cell growth, volumetric ALP production, and substrate consumption profiles are shown in Fig. 7. The cell growth and enzyme production patterns were similar to those obtained in shake-flask cultivation, although lower. The cell mass grew exponentially to the biomass production peak (*X*_*max*_ = 5.06 g L^–1^) at 24 h, which is approximately 41.13% lower than that obtained from shake-flask and uncontrolled pH batch cultivation. The exponential growth rate and yield coefficient *Y*_*x/s*_ were 0.122 g L^–1^ h^–1^ and 0.23 (g of cells/g of the substrate), respectively, which are lesser than those achieved from shake-flask and uncontrolled pH batch cultivation by a factor of 0.315 and 0.2875, respectively. Moreover, the specific growth rate (*µ* = 0.0331 h^–1^) was lower than that obtained from shake-flask and uncontrolled pH batch cultivation by a factor of 0.464 and 0.350, respectively.

Similarly, the volumetric productivity of ALP increased gradually throughout the fermentation period to its maximum at 20 h of 2175.5 U L^–1^, representing a decrease from that obtained from shake-flask and uncontrolled pH batch cultivation by a factor of 2.06 and 3.08, respectively, as shown in Fig. 7a. The yield coefficient *Y*_*p*/x_ (536.19 U g^–1^ of the biomass) is approximately 1.35 times that obtained via shake-flask cultivation but 0.514 times lower than that obtained from the uncontrolled pH batch cultivation. The production rate (*Q*_*p*_) and yield coefficient *Y*_*p/*s_ were both lower than in shake-flask or uncontrolled pH batch cultivation and estimated as 121.71 U L^–1^ h^–1^ and 98.55 U g^–1^, respectively. The total carbohydrate and phosphate consumption similarly showed a more-gradual decline, reaching concentrations of 0.997 and 0.007 g L^–1^, respectively, at the end of cultivation (28 h) with overall consumptions of 0.817 and 0.0012 g L^–1^ h^–1^. This decrease in total carbohydrate and phosphate consumption was due to the reduction in cell growth and enzyme productivity. The dissolved oxygen concentration decreased rapidly to 1.8% after 2 h of cultivation, reached a zero concentration after 7 h of cultivation, and remained there until the end of fermentation, as shown in Fig. 7b.

Controlling the pH decreased the growth of *Bacillus paralicheniformis* strain APSO and presented obstacles for cell growth and thus inhibited the productivity of ALP. The uncontrolled pH batch cultivation is thus deemed the most suitable and favored condition for promoting and enhancing the productivity of ALP. Overall, the productivity of ALP derived from *Bacillus paralicheniformis* strain APSO was systematically improved through PBD (1475.83 U L^–1^), RCCD (4488.266 U L^–1^), and an uncontrolled pH batch cultivation strategy (6650.92U L^–1^) by factors of 20.96, 70.12, and 94, respectively, when compared with the basal medium (70.4 U L^–1^). Due to the scarcity of literature available, a comparison of the presented results with those of prior researchers is difficult. This work thus represents the first report concerning the scaling-up of the production of extracellular ALP from *Bacillus paralicheniformis* strain APSO to a benchtop bioreactor scale using biogenic apatite and sugarcane molasses as nutrient sources for stimulating ALP production.

### Morphological structure of the animal bone powder

**Figure 8:**
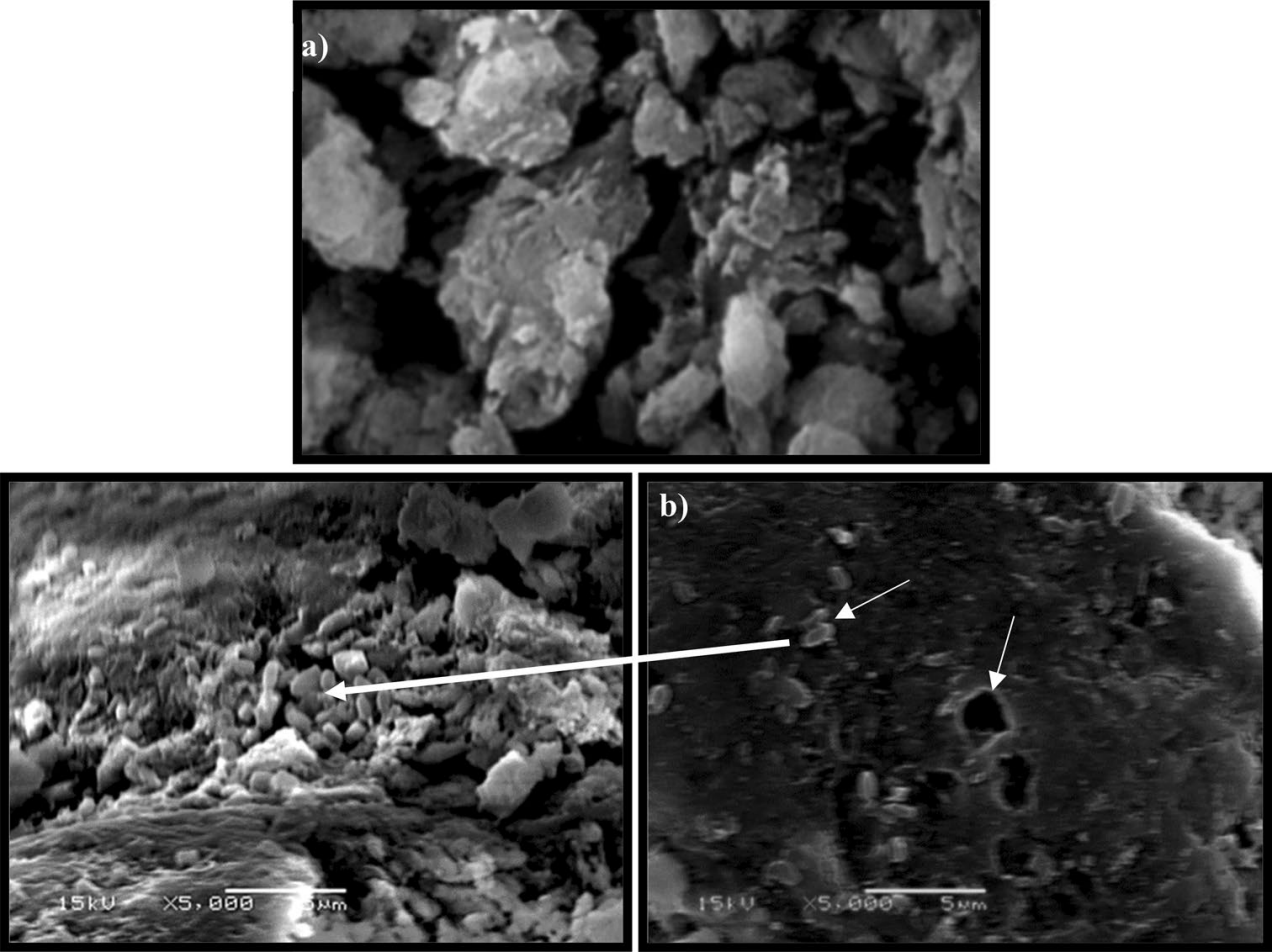
Scanning electron microscopy (SEM) micrograph of the animal bone powder of the (**a**) untreated (control) sample and (**b**) treated sample after fermentation showing the morphological surface of the bone at a magnification of 5000 with 15 kV.

The residual animal bone powder was collected after fermentation by filtering the bacterial culture through Whatman #1 filter paper and drying it overnight in an oven at 60 °C. The collected powder was then subjected to SEM and compared with an untreated sample; the obtained micrographs are shown in Fig. 8. In the treated sample, there is obvious degradation due to the bacterial ALP, whereas the untreated sample had a smooth surface. As previously documented^5^, biogenic apatite is rich in calcium phosphate and contains fewer impurities (e.g., fluorine) than mined and beneficiated rock phosphate, making bone a more reactive form of phosphorus than other phosphate counterparts.

### Fourier-transform infrared spectroscopy (FT-IR) analysis

**Figure 9:**
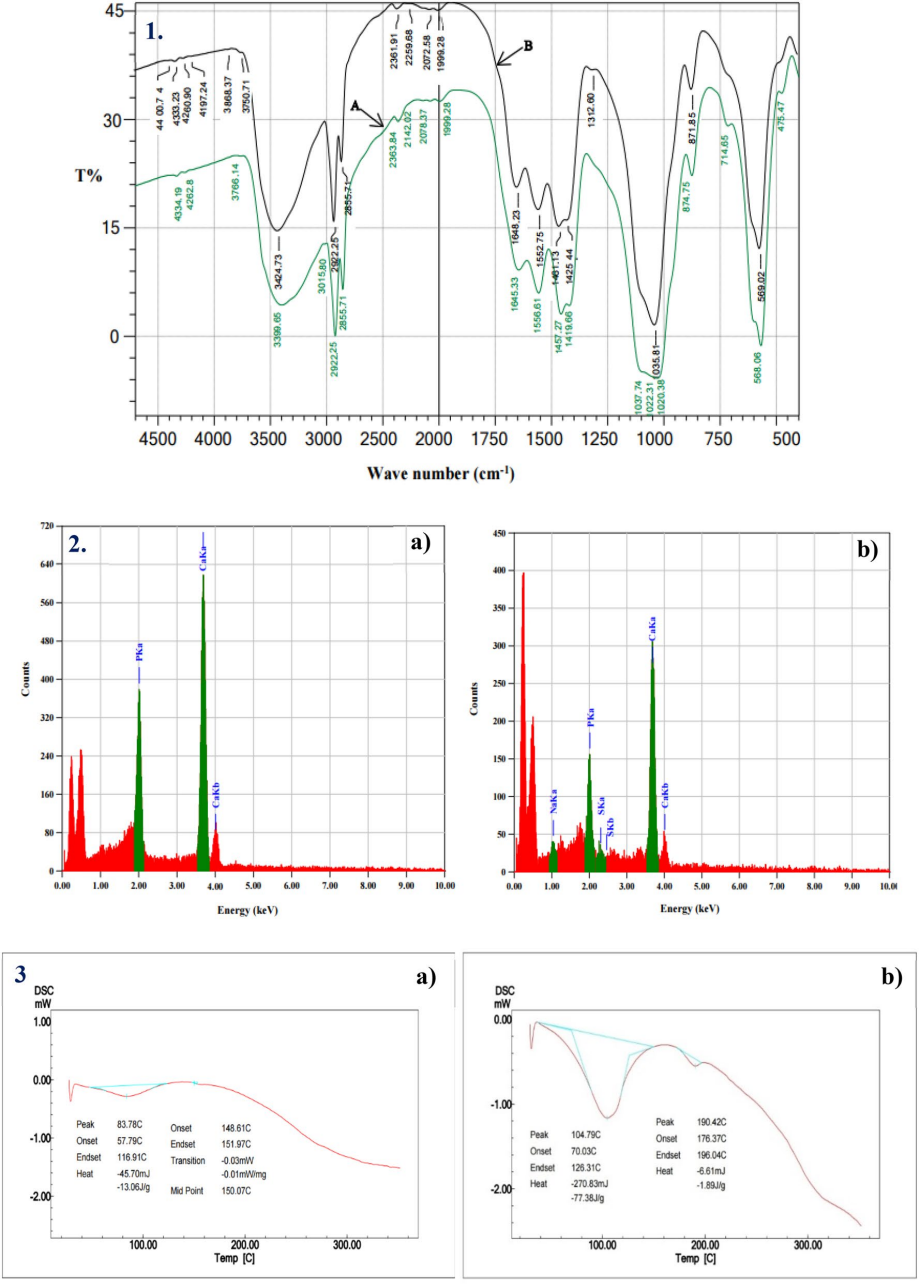
Physicochemical properties of animal bone powder: (**1**) FTIR pattern of the (A) untreated (blank) sample and (B) treated sample after fermentation; (**2**) EDX analysis of the (**a**) untreated (blank) sample and (**b**) treated sample after fermentation; and (**3**) DSC analysis pattern of the (**a**) untreated (blank) sample and (**b**) treated sample after fermentation.

The FT-IR spectrums of animal bone powder samples before and after fermentation, shown in Fig. 9(1), were analyzed to detect any differences due to the action of ALP produced by *Bacillus paralicheniformis* strain APSO on the bone. Before fermentation, the biogenic apatite sample showed characteristic absorption peaks at 3399, 2363, 2142, 2078, 1645, 1556, 1457, 1419, 1037, 874, and 568 cm^−1^; after fermentation, these peaks were shifted to 3424, 2361, 2259, 2072, 1648, 1552, 1461, 1425, 1035, 871, and 569 cm^−1^, respectively. These changes in the wavenumbers and their intensity were a result of the interaction between the functional groups of secreted metabolites and bone during fermentation that modified the existing functional groups on the surface of the bone.

The broad peak observed at 3424.7 cm^−1^ in the treated bone sample was assigned to the O–H stretching vibrations that occur within a broad range of frequencies, thus indicating the presence of free hydroxyl groups and the bonded O–H peaks of carboxylic acids. The stretching vibration bands at 2922.3 and 2259 cm^−1^ were related to the C–H stretch representing alkane groups and C≡C medial alkyne (disubstituted), respectively, whereas the peak at 2072 cm^−1^ can be assigned to the N=C=S (isothiocyanate) group. The vibration bands at 2361, 1648.2, and 1552.8 cm^−1^ were assigned to the N–H stretch of the amino-related component, amide I (C–O stretch), and amide II (N–H in-plane bending), respectively, whereas the vibration band at 1461.1 and 1425.4 cm^–1^ represents CH_2_ bending and CH_3_ (methyl C–H asym/sym bend), respectively. The latter finding is in agreement with that by Roguska et al.^27^, who documented that the vibration bands at approximately 1650 and 1535 cm^−1^ are related to amide I and amide II, respectively. Two strong peaks observed at 1035.8 and 569 cm^−1^ were assigned to the asymmetric deformation vibration of P=O in PO_4_^3^ as a result of ALP action, as Manalu et al.^28^ found the phosphate group to be strong at 1030–1090 and 560 cm^–1^. Additionally, the vibration band present at 871.9 cm^–1^ in the spectrum of the treated bone powder is the characteristic absorption peak of calcite (CO_3_^−2^) vibrations according to Hosseinzadeh et al.^29^, who showed that the carbonate group is located at 873 cm^−1^.

### Energy-dispersive spectroscopy (EDS) analysis

Energy-dispersive spectroscopy (EDS) was used for the elemental analysis of the biogenic apatite; the resulting analyses of untreated and treated bone powder are shown in Fig. 9(2a) and (2b), respectively. In the untreated sample, two absorption peaks corresponding to phosphate were present with an atomic and mass percentage of 25.67% and 21.07%, respectively, as was a peak corresponding to calcium with an atomic and mass percentage of 74.33% and 78.93%, respectively, thus confirming the typical elemental composition of bone. After fermentation, the atomic and mass percentage of phosphate decreased to 19.07% and 15.89%, respectively, and the atomic and mass percentage of calcium decreased to 72.85% and increased to 78.52%, respectively. This increase in the mass percentage of calcium and decrease in the phosphate content was due to the production of ALP by *Bacillus paralicheniformis* strain APSO, during which organic calcium phosphate is hydrolyzed to inorganic soluble phosphate, which is then consumed by a bacterial cell for growth and production. Thus, the Ca:P weight ratio of the biogenic apatite fermentation reduced from 2.89 before fermentation to 3.82 after fermentation. Additionally, traces of sodium and sulfur were appeared, having an atomic percentage of 5.58% and 2.49%, respectively, and a mass percentage of 3.45% and 2.15, respectively.

### Differential scanning calorimetry (DSC) analysis

Differential scanning calorimetry (DSC) was used to estimate the qualitative and quantitative thermal properties of the biogenic apatite; the resulting thermograms of the apatite before and after fermentation are shown in Fig. 9(3a) and (3b), respectively. After fermentation, the primary endothermic transition of the bone powder presented a melting temperature peak at 104.79 °C, heat flow of – 1. 17 mW mg^–1^, the heat capacity of – 270.83 mJ, and enthalpy of – 77.38 J g^–1^; the second degradation temperature peak at 190.42 °C presented a heat flow of – 0.55 mW mg^–1^, the heat capacity of – 6.61 mJ, and enthalpy of – 1.89 J g^–1^. These peaks are due to the ALP-induced hydrolysis of organic phosphate (from calcium phosphate to soluble inorganic phosphate), making the bone easier to melt and degrade. These results are in good agreement with those obtained by Manalu et al.^27^, who found via DSC analysis that the two inflection peaks of bovine bone at 100.5 °C and 350 °C corresponded to the removal of water and organic matter, respectively.

## Conclusion

This work presented novel strategies for the statistical optimization and scaling up of ALP production by a local bacterial isolate exhibiting ALP production that was isolated from a black liquor sample obtained from a local paper and pulp factory and was identified as *Bacillus paralicheniformis* strain APSO through molecular and morphological characterization. To increase the productivity of ALP, sequential mathematical optimization and bioprocessing scaling up approaches were applied using low-cost nutrient sources such as biogenic apatite (i.e., waste animal bone powder) and sugarcane molasses as ALP stimulators. The use of fractional-factorial PBD allowed for a 20-fold increase in the productivity of ALP over that using an initial basal medium and demonstrated that molasses, (NH_4_)_2_NO_3_, and KCl were the most significant factors influencing ALP productivity. Applying RCCD to clarify the correlation among the variables most significantly affecting throughput via a polynomial quadratic model then allowed for an overall ALP productivity improvement by a factor of 70 over the original basal medium, thus demonstrating the ability to enhance productivity using the proposed sequential statistical optimization strategies. Batch cultivation was then scaled up to a 7-L benchtop bioreactor under controlled and uncontrolled pH conditions to evaluate the scalability and kinetics of microbial growth in a submerged cultivation system. When the pH was not controlled, ALP production increased during the exponential growth of *Bacillus paralicheniformis* strain APSO, and a 94-fold increase in the volumetric productivity of ALP was found over that obtained by the initial basal medium. However, under pH-controlled conditions, neither the growth of *Bacillus paralicheniformis* strain APSO nor the productivity of ALP was supported. Overall, this novel approach will provide an innovative and efficient production strategy of bacterial alkaline phosphatase to meet present and forecast market demand.

## Material and methods

### Sample collection and isolate sources

The bacterial strain under investigation, *Bacillus paralicheniformis*, was isolated from black liquor samples obtained aseptically from Alexandria paper and pulp manufactories. The samples were transported to the Bioprocess Development Department laboratory and stored in the refrigerator at 4 °C until further processing.

### Enrichment and isolation of ALP-producing bacteria

The ALP-producing bacteria were enriched in a modified PVK broth medium with a pH of 7.0 containing, in g L^−1^: glucose, 10; (NH_4_)_2_SO_2_, 0.5; NaCl, 0.5; yeast extract, 0.5; KCl, 0.2; MgSO_4_·7H_2_O, 0.1; MnSO_4_.H_2_O, 0.002; FeSO_4_.7H_2_O, 0.002; and animal bone powder, 5, rather than Ca_3_(PO4)_2_^29^. The animal bone powder was obtained from glue factories (Borg Al-Arab, Alexandria, Egypt) and passed through a 0.125-mm sieve.

1.0 ml of black liquor was suspended in a 250-mL Erlenmeyer flask containing 50 ml of the modified PVK broth medium; this mixture was incubated at 50 °C under shaking (200 rpm) for 72 h. The enriched cultivated sample was then serially diluted using a sterile saline solution as a diluent. From these diluted samples, 50 µl aliquots were plated into the sterile Luria–Bertani (LB; in g L^−1^: peptone, 10; NaCl, 10; yeast extract, 5) medium agar plate. The inoculated plates were incubated overnight at 45 °C. The isolated colonies were further purified under aseptic conditions using the continuous streaking method. Pure isolates were subcultured, maintained on LB slants, stored at 4.0 °C, and sub-cultured regularly.

### Qualitative screening for ALP activity

The pure isolates were screened for the production of extracellular phosphatase production using two kinds of selective media agar, MG-PDP, and pNPP, both of which contained, in g L^−1^: glucose, 1; peptone, 10; NaCl, 10; and yeast extract, 5. The MG-PDP medium was supplemented with 50 µg mL^−1^ methyl green dye and 1 mg/ml PDP (Sigma)^30^, whereas the pNPP medium was supplemented with chromogenic substrate 1 mg mL^−1^ pNPP as a screening media^31^. The pure cultures were streaked or pipetted out onto the well at the center of the sterile screening medium and the plates were incubated at 45 °C overnight. The appearance of green- or yellow-stained colonies indicated ALP production. A promising isolate showing the highest color intensity was designated as PL3 and was subjected to further study via morphological and molecular identification.

### Quantitative screening for ALP activity

To quantitatively assess the activity of the ALP, 50 mL of modified PVK broth dispensed in a 250 mL Erlenmeyer flask was inoculated with one ml of a 12-h-old suspension of the selected isolate and incubated at 45 °C for 72 h using a rotary shaker (200 rpm). The cell-free supernatant was harvested after incubation by centrifugation at 6000 rpm for 20 min under cooling (4 °C). The activity was measured colorimetrically by monitoring the release of *p*-nitrophenol from *p*-nitrophenyl phosphate disodium salt (*p*NPP, Sigma) as a chromogenic substrate with the molar extinction coefficient (ε) 405 = 18,000 M^–1^ cm^–1^ at 405 nm^32^. A typical reaction mixture contained appropriately diluted enzyme solution in 250 µL of 0.1 M modified universal buffer (MUB), pH 10.5; 600 µL of 0.1 M modified universal buffer, pH 10.5; and 250 µL of *p-*NPP reagent (10 mM). The reaction mixture was incubated after thoroughly mixing at 70 °C for 3 min. The reaction was terminated by adding 50 µL of 4.0 M NaOH. The blank contained all constitutes except the active enzyme. The amount of *p*-nitrophenol released from *p*-NPP was monitored at 405 nm (ε = 18,000 M^–1^ cm^–1^) using a UV–Vis spectrophotometer. One unit of enzyme activity has been expressed as an international unit (U), which represents the amount of enzyme that catalyzes the hydrolysis of 1.0 μmol of *p*NPP to *p*-nitrophenol in one minute at pH 10.5 and 70 °C; the activities were expressed in U L^–1^ min^–1^.

### Amplification of the 16S rDNA gene, sequencing, and similarity

The genomic DNA of the potential ALP-producing isolate was extracted by the salting-out method^33^. The PCR reaction and sequencing were executed according to the method detailed by Abdelgalil et al.^34^.

### Morphological investigation of *Bacillus paralicheniformis* strain APSO

SEM (JEOL JSM 6360 LA, Japan) was performed at the laboratory center at the City of Scientific Research and Technological Applications (SRTA-City) to visualize the morphology of the cell surface of the most potent ALP-producing bacteria. A thin film of a 12-h-old culture of the strain under investigation was taken on a coverslip, air-dried, and fixed by flamming. The dried bacterial thin film was coated with a thin layer of gold using a sputtering device (JFC-1100 E JOEL, USA) for 12 min. A micrograph at 20 kV was then obtained.

### Effect of some of the physical parameters on ALP productivity

The efficiency of the productivity of the ALP was investigated for various physical parameters such as temperature, pH, and initial inoculum size. To determine the effective temperature for ALP production by the selected isolate, 50 mL of modified PVK broth^29^ separately dispensed in a 250 mL Erlenmeyer flask and sterilized was inoculated with a standard inoculum 1.0-mL aliquot of activated pre-culture and was incubated at different temperatures (40 °C, 45 °C, and 50 °C) for 72 h using a rotary shaker (200 rpm); 2.0-ml aliquots were drawn out to measure the activity of the ALP. Different initial pH values (7, 8, 9, and 10) were examined to characterize their influence on ALP production. The isolate under investigation was cultivated in a 250 mL Erlenmeyer flask containing 50 mL modified PVK broth at each pH value by a standard inoculum of activated pre-culture (1.0 mL). The pH of the medium was adjusted by dissolving the medium constituents in 0.1 M Tris-NaOH buffer for pH values of 7.0, 8.0, and 9.0 and in 0.1 M glycine–NaOH buffer for a pH value of 10.0. The initial inoculum size was estimated by using 2%, 5%, and 10% of 12-h activated pre-culture of the isolate of interest.

### Statistical optimization for ALP production

Recently, statistical experimental designs have been regarded as powerful tools to overcome obstacles, since the significant variables can be checked and studied for complex interactions^35^.

### Plackett–Burman design (PBD)

The statistical experimental methodology designed by Plackett and Burman (PBD) is an efficient screening technique that identifies the most significant variables from an array of factors and their main effects; however, the effects of their interactions are not considered^36^. A fractional-factorial PBD with three central points was exploited to determine which of the eight factors considered significantly influenced the productivity of ALP by using the identified *Bacillus paralicheniformis* strain APSO in submerged fermentation. According to this design, fifteen trial batches were developed (twelve main batches and three central-point batches). The independent variable was studied at two widely spaced levels, coded as high (+ 1) and low (–1), which were used to define the upper and lower limits of the range covered by each variable, respectively. The list of the variables studied and their corresponding coded and actual levels are shown in Table 1, as well as the matrix layout. Accordingly, the independent variables were screened in fifteen combinations using the coded level of each variable; the corresponding response in terms of ALP yield was then obtained. As all experiments were performed in duplicate, the average ALP productivity was taken as the response (*Y*), which was based on the first-order polynomial model and calculated as:

3 $$Y_{{\text{ALP activity}}} = \beta_{{\text{o}}} + \, \sum \beta_{i} X_{i} ,$$

where *Y* is the predicted response (i.e., the ALP activity, in U L^–1^ min^–1^), *β*_*o*_ is the model intercept, *β*_*i*_ is the linear regression coefficient, and *X*_*i*_ is the coded independent variable estimate. The effect of the individual variable on ALP productivity was calculated using the following equation.

4 $$E\left( {X_{i} } \right) = { 2 }(\Sigma M_{H} {-}M_{L} )/N,$$

where *E*(*X*_*i*_) is the response effect of variable *X*_*i*_; *M*_*H*_ and *M*_*L*_ represent the efficiency of ALP productivity obtained at the high and low levels for each variable, respectively; and *N* is the total number of trials (*N* = 15). The essential experimental design free download software programs were exploited for the statistical analysis, determination of coefficients as well as polynomial model reduction. To evaluate the efficiency and effectiveness of the regression model, ANOVA was conducted and the values of R and R^2^ were calculated.

The variables with confidence levels above 95% and *p*-values below 0.05 (*p* < 0.05) were considered to significantly influence ALP production and were selected for further optimization by RCCD^37^. The predicted optimum levels of the independent variables were analyzed; they were then compared to the basal condition setting and the average enzyme production was calculated.

### Response surface methodology (rotatable central composite design)

The three parameters with the highest confidence levels (molasses, (NH_4_)_2_NO_3_, and KCl) were left to be further optimized after the PBD. To describe the nature of the response surface in the experimental region and evaluate the optimum levels of the significant variables and interactions between the selected variables with high influence on the productivity of ALP, RCCD was applied. The five-level, three-factorial RCCD method was employed to fit the polynomial model and the experimental errors were calculated based on the standard deviation of the center point with four runs. Each of the independent variables was studied at five different levels, − α, − 1,0, + 1, and + α, where α = 1.6817^38^, while the insignificant variables were held at their zero points (center value). All trials were conducted in duplicate and the mean duplicates values (alkaline productivity) were taken as responses. The coded and actual level of the significant variables under investigation and the layout of the experimental design matrix are summarized in Table 3. According to the corresponding RCCD design, combinations of the three independent significant variables were conducted in eighteen experiments; data analysis was performed via quadratic regression to clarify the main and interaction effects of the significant independent factors according to the response value maximization under the optimal concentration of each parameter. The following quadratic equation may convey factors as a function of response values:

5 $${\text{Y }} = \, \beta_{0} + \Sigma_{{\text{i}}} \beta_{{\text{i}}} {\text{X}}_{{\text{i}}} + \Sigma_{{{\text{ij}}}} \beta_{{{\text{ij}}}} {\text{X}}_{{\text{i}}} {\text{X}}_{{\text{j}}} + \Sigma_{{{\text{ii}}}} \beta_{{{\text{ii}}}} {\text{X}}_{{\text{i}}}^{{2}} ,$$

where *X*_*i*_ and *X*_*j*_ represent the independent variables and *β*_*ij*_ and *β*_*ii*_ represent the cross-product and quadratic coefficients, respectively. Laboratory validation was performed to evaluate the equation model and ensure that the theoretical values of each variable were computed accurately. The three-dimensional response surface plots were planned to determine the interaction among the significant variables and the point-prediction approach was used to determine the optimal values for each variable via software trial version of STATISTICA 7.0. The obtained data were analyzed via multiple linear regression using the essential experimental design free download software. The significance of variables was calculated by ANOVA and judged statistically by computing the F-value at a probability (*p*-value) of < 0.05. The multiple coefficients of correlation (R^2^) and the adjusted determination coefficient (R^2^adj) were calculated to assess the validity of the model.

### Scale-up production of bacterial ALP

Bioreactors, often regarded as the heart of the fermentation process, have helped researchers overcome issues facilitating the scale-up of submerged fermentation from a shake flask to production scale^39^. As this work aims to establish a large-scale fermentation system to evaluate the kinetics of microbial growth in a submerged cultivation system, both shake-flask and stirred-bioreactor batch cultivation methodologies were employed and compared.

### Shake-flask batch cultivation

Shake-flask batch fermentation was conducted by inoculating a 250-ml Erlenmeyer flask containing 50 mL of the optimized medium (containing, in w/v%: molasses, 30.0; (NH_4_)_2_NO_3_, 0.795; NaCl, 0.5; MgSO_4_·7H_2_O, 0.11; animal bone powder, 0.1; KCl, 1.213; CoCl_2_·6H_2_O, 0.0025; and MnSO_4_·H_2_O, 0.0005; the medium ingredients were dissolved in a 0.1 M Tris-NaOH buffer to maintain a pH of 9.0 ± 2) with 10-h-activated pre-cultured inoculum (5%) and incubated in an orbital shaker at 45 °C and 200 rpm. At regular intervals of the incubation period, the samples were drawn out periodically every 2 h. A Beckman DU spectrophotometer was used to measure the absorbance at 600 nm against a blank (optimized medium without inoculation). Aliquots (5 mL) of the bacterial culture were centrifuged at 10,000 rpm for 10 min under cooling conditions 4 °C to obtain cell-free supernatants and then kept in the refrigerator for further analysis. The ALP activity, total soluble phosphate concentration, total carbohydrate concentration, and total soluble protein concentration were monitored constantly at different time intervals of the incubation period. All experiments were carried out in triplicate.

### Stirred-bioreactor batch cultivation system

A 7-L benchtop bioreactor with a working volume of 4.0 L (Bioflow 310, New Brunswick, NJ, USA) equipped with two 6-bladed disk-turbine impellers and four baffles was used for batch cultivation. A Bio-Command multi-process control software supported by a 10.4 color touch-screen control panel was used to automate the process. The bioreactor was equipped with an air compressor to supply compressed air at 0.5 VVM (air volume per broth volume per minute) via a sterile filter; additionally, a digitally controlled pH electrode, a temperature probe, and polarographic dissolved oxygen (DO) electrode (Ingold, Mittler-Toledo, Switzerland) were installed. Uncontrolled-pH batch fermentation was initiated by inoculating the bioreactor vessel containing 3800 mL of the optimized sterile medium with 5% of the log-phase activated pre-cultured inoculum aseptically. The fermentation temperature, agitation speed, and pH were set to 45 °C, 200 rpm, and ± 9.0, respectively; the pH was controlled by automatic feeding of 2 M NaOH and 2 M HCl. An antifoaming agent (silicone oil, 0.5:10 v/v) was added at a concentration of 1:100 (v/v) in distilled water to suppress the occurrence of foam throughout the fermentation process. Periodically at different time intervals, 20-ml culture samples were withdrawn in pre-weighed, 50-ml sterile falcon tubes to track the cell growth via spectrophotometry at 600 nm against a blank using a Beckman DU spectrophotometer. The cell-free supernatant was obtained by centrifugation at 6000 rpm for 15 min and used for further analytical procedures, whereas the collected cell pellets were used to determine the dry weight of the cell biomass.

### Analytical procedures

#### Determination of biomass dry weight

The biomass dry weight was determined gravimetrically by collecting 10-ml bacterial cultures in pre-weighed 15-ml sterile falcon tubes at different time intervals during the fermentation process and centrifuging them at 10,000 rpm for 10 min to harvest cell pellets. The cell pellets were then washed twice with distilled water, centrifuged, and dried to a constant weight at 70 °C. To estimate the correlation factor (*δ*), the linear relationship between the biomass dry weight and the optical density (OD_600_) of the bacterial culture was constructed.

#### Determination of total carbohydrates concentration

The concentration of total carbohydrates in the culture filtrate samples was analyzed spectrophotometrically using an anthrone reagent; the developed greenish color was measured quantitatively against a sample-free blank at 620 nm, and 0.1 mg mL^−1^ of stock sucrose solution was used for plotting the standard curve^40^.

#### Determination of total soluble phosphate content.

Chen’s method^41^ was used to spectrophotometrically estimate the available phosphorus in the culture filtrate. The intensity of the molybdenum-blue color measured at 820 nm and the standard curve were plotted using 100 µg mL^−1^ of stock potassium dihydrogen phosphate solution to determine the concentration.

#### Protein concentration assay

Protein contents were estimated by Lowry’s method^42^ and the absorbance was determined at 660 nm using bovine serum albumin (BSA, Sigma) as a standard.

#### Atomic absorption analysis

Atomic absorption spectrometry (Analytik Jena, Zeenit 700, Germany) was performed at the laboratory center of the SRTA-City to determine the residual concentrations of heavy metals in the culture filtrate via the standard method^43^.

#### Morphological structure of the animal bone powder

The surface morphology of the animal bone powder before and after submerged cultivation of the bacterial cell was characterized using SEM at the laboratory center of the SRTA-City.

#### Fourier-transform infrared spectroscopy (FT-IR)

To investigate the functional groups and chemical bonds of animal bone powder before and after the fermentation process, 1-mg samples containing 100 mg of KBr were ground up, compressed into a transparent disk, and analyzed using Fourier-transform infrared spectrophotometry (Shimadzu FTIR-8400S, Japan) in the mid-IR range between 4000 and 400 cm^–1^ at the laboratory center of the SRTA-City.

#### EDS analysis

Animal bone powder samples were examined by SEM (JSM-6700F, JEOL, Japan) equipped with an in-situ EDS spectrophotometer.

#### DSC analysis

The residual animal bone powder samples before and after fermentation were collected by filtering the bacterial culture using Whatman #1 filter paper and then dried in an oven overnight at 60 °C; the collected powder was mounted in an aluminum sample pan and subjected to DSC (60–A) to estimate its pyrolysis pattern. The analysis was performed in a nitrogen atmosphere with a heating rate of 10 °C min^−1^ and a flow rate of 30 mL min^−1^. The thermogram was obtained from 25 to 350 °C and plotted as temperature versus heat flow.

## Data Availability

All data produced during this study are included in this published article.

## References

[1] Aguilar A, Wohlgemuth R, Twardowski T (2018). Perspectives on bioeconomy. New Biotechnol..

[2] Et SM, Mohamed CN (2017). A Sustainable Bioeconomy: The Green Industrial Revolution.

[3] Scarlat N, Jean-François D, Fabio M, Viorel N (2015). The role of biomass and bioenergy in a future bioeconomy: Policies and facts. Environ. Dev..

[4] Alcalde M, Manuel F, Francisco JP, Antonio B (2006). Environmental biocatalysis: From remediation with enzymes to novel green processes. Trends Biotechnol..

[5] Saeid A, Labuda M, Chojnacka K, Górecki H (2014). Valorization of bones to liquid phosphorus fertilizer by microbial solubilization. Waste Biomass Valoriz..

[6] Rasmey AM, Heba HH, Omar AA, Akram AA (2018). Enhancing bioethanol production from sugarcane molasses by *Saccharomyces cerevisiae* Y17. Egypt J. Bot..

[7] Sharma B, Arun KD, Pratyoosh S (2018). Contemporary enzyme-based technologies for bioremediation: A review. J. Environ. Manage..

[8] Pandey SK, Banik RM (2012). Selection and optimization of nutritional constituents for enhanced production of alkaline phosphatase by *Bacillus licheniformis* MTCC 1483. J. Agric. Technol..

[9] Alami NH (2019). Extracellular alkaline phosphatase from mangrove soil yeast. Indonesia Chim. Acta..

[10] Patel FR (2016). Purification and characterization of alkaline phosphatase from a halotolerant facultative alkaliphile *Bacillus flexus* FPB17. Int. J. Pharm. Sci. Res..

[11] Kannaiyram S, Vedhachalam R, Thanigaimalai M (2015). Production and characterization of alkaline phosphatase produced by *Bacillus* Species. J. Appl. Biol. Biotechnol..

[12] Singh V (2017). Strategies for fermentation medium optimization: an in-depth review. Front. Microbial..

[13] Jinendiran S (2019). Optimization of submerged fermentation process for improved production of β-carotene by *Exiguobacterium acetylicum* S01. Heliyon..

[14] Patel Falguni R, Sharma MC (2014). Novel organophosphate pesticide utilizing alkaline phosphatase producing polyextremophile bacillus flexus from lake ecosystem of North Gujarat, India. Int. J. Innov. Res. Sci. Eng. Technol..

[15] Pandey SK, Banik RM (2010). Optimization of process parameters for alkaline phosphatase production by *Bacillus licheniformis* using response surface methodology. Int. J. Agric. Technol..

[16] Jatoth K, Shantipriya A, Mangilal T, Junapudi S (2015). Optimization for the production of extracellular alkaline phosphatase from *Bacillus subtilis*. Int. J. Curr. Microbiol. Appl. Sci..

[17] Behera B (2017). Alkaline phosphatase activity of a phosphate solubilizing *Alcaligenes faecalis*, isolated from Mangrove soil. Biotechnol. Res. Innov..

[18] Chaudhuri, G., Selvaraj, U., Babu, V. & Thilagaraj, R. W. Recent Trends in Phosphatase-Mediated Bioremediation, In *Phosphoric Acid Industry: Problems and Solutions*. 27–46 (In Tech, 2017).

[19] Priya D, Kumar DJM, Kalaichelvan PT (2014). Optimization and production of extracellular alkaline phosphatase from *Bacillus megaterium*. Int. J. Chemtech. Res..

[20] Montgomery, D. Experiments with a single factor: The analysis of variance. In: Design and analysis of experiments. 87–89, (John Wiley and Sons, 1991).

[21] Qureshi A, Muhammad UD, Sartar IP (2010). Biosynthesis of alkaline phosphatase by *Escherichia coli* EFRL 13 in submerged fermentation. World Appl. Sci. J..

[22] Behera SK, Himanshu M, Sudipto C, Meikap BC (2018). Application of response surface methodology (RSM) for optimization of leaching parameters for ash reduction from low-grade coal. Int. J. Min. Sci. Technol..

[23] Nwabueze TU (2010). Basic steps in adapting response surface methodology as mathematical modelling for bioprocess optimisation in the food systems. Int. J. Food Sci. Technol..

[24] Ali S, Syeda TA, Syeda FN, Muneeba S (2018). Strategies and kinetics of industrial fermentation for the mass production of various primary and secondary metabolites from microbes. Eur. J. Pharm. Med. Res..

[25] Butler AJ, Hallett DS, Macaskie LE (1991). Phosphatase production by a *Citrobacter* sp. growing in batch culture and use of batch cultures to investigate some limitations in the use of polyacrylamide gel-immobilized cells for product release. Enzyme Microb. Technol..

[26] Chaudhuri, G., Dey, P., Dalal, D., Venu-Babu, P. & Thilagaraj, W. R. A novel approach to precipitation of heavy metals from industrial effluents and single-ion solutions using bacterial alkaline phosphatase. *Water Air Soil Pollut*. **7**, 1625 (2013).

[27] Roguska A (2011). Characterization of calcium phosphate–TiO2 nanotube composite layer for biomedical applications. Mater. Sci. Eng. C. Mater..

[28] Manalu J, Bambang S, Decky JI (2015). Characterization of hydroxyapatite derived from bovine bone. Asian J. Appl. Sci..

[29] Hosseinzadeh E (2014). Fabrication of a hard tissue replacement using natural hydroxyapatite derived from bovine bones by thermal decomposition method. Int. J. Organ. Transplant. Med..

[30] Parhamfar M, Badoei-Dalfard A, Parhamfar M, Rad SF (2016). Purification and characterization of an extracellular phosphatase Enzyme from *Bacillus* spp. J. Cell Mol. Res..

[31] Kulkarni S, Chitra SM, Alka G, Anand B, Shree KA (2016). Interaction of uranium with bacterial cell surfaces: Inferences from phosphatase-mediated uranium precipitation. Appl. Environ. Microbiol..

[32] Hashem KA, Sawsan HA, Luqaa HM (2016). In vivo antibacterial activity of alkaline phosphatase isolates from *Escherichia coli* isolated from diarrhea patients against *Pseudomonas aeruginosa*. J. Pharm. Innov..

[33] Obidi OF, Olushina OA, Miriam NI, Foluke OO (2018). Production of phosphatase by microorganisms isolated from discolored painted walls in a typical tropical environment: A non-parametric analysis. Arab. J. Basic Appl. Sci..

[34] Marahatta SB (2006). Polymorphism of glutathione S-transferase omega gene and risk of cancer. Cancer Lett..

[35] Abdelgalil A, Morsi A, Reyed M, Soliman A (2018). Application of experimental designs for optimization the production of *Alcaligenes faecalis* Nyso Laccase. J. Sci. Ind. Res..

[36] Yong X (2011). Optimization of the production of poly-γglutamic acid by *Bacillus amyloliquefaciens* C1 in solid-state fermentation using dairy manure compost and monosodium glutamate production residues as basic substrates. Bioresour. Technol..

[37] Plackett RL, Burman JP (1946). The design of optimum multifactorial experiments. Biometrika.

[38] El-Naggar NEA, Hamouda RA, Mousa IE, Abdel-Hamid MS, Rabei NH (2018). Biosorption optimization, characterization, immobilization and application of *Gelidium amansii* biomass for complete Pb^2+^ removal from aqueous solutions. Sci. Rep..

[39] Jinendiran S, Kumar BD, Dahms HU, Arulanandam CD, Sivakumar N (2019). Optimization of submerged fermentation process for improved production of β-carotene by *Exiguobacterium acetylicum* S01. Heliyon..

[40] Deljou A, Iman A, Morteza K (2018). Scale-up thermostable α-amylase production in lab-scale fermenter using rice husk as an elicitor by *Bacillus licheniformis*-AZ2 isolated from Qinarje Hot Spring (Ardebil Prov. of Iran). Period Biol..

[41] Morris DL (1948). Quantitative determination of carbohydrates with Dreywood's anthrone reagent. Science.

[42] Chen PS, Toribara TY, Warner H (1956). Micro-determination of phosphorus. Anal. Chem..

[43] Lowry OH, Rosebrough NJ, Farr AL, Randall RJ (1951). Protein measurement with the folin phenol reagent. J. Biol Chem..

[44] Baird, R. & Bridgewater, L. Standard methods for the examination of water and wastewater. (American Public Health Association, 2017).

