# Author Correction: Dynamic consolidated bioprocessing for innovative lab-scale production of bacterial alkaline phosphatase from *Bacillus paralicheniformis* strain APSO

**DOI:** 10.1038/s41598-021-95272-4

**Published:** 2021-08-03

**Authors:** Soad A. Abdelgalil, Nadia A. Soliman, Gaber A. Abo-Zaid, Yasser R. Abdel-Fattah

**Affiliations:** 1 Bioprocess Development Department, Genetic Engineering and Biotechnology Research Institute (GEBRI), City for Scientific Research and Technological Applications, Alexandria, Egypt; 2 Present Address: New Borg El-Arab City, Universities and Research Institutes Zone, Post Alexandria, 21934 Egypt

Correction to: *Scientific Reports* 10.1038/s41598-021-85207-4, published online 16 March 2021

The original version of this Article contained an error in the legend of Figure 1

“Schematic illustration the utilization of biogenic apatite waste to scale up the production of bacterial ALP from the *Bacillus paralicheniformis* strain APSO to the benchtop bioreactor scale.”

now reads:

“Schematic illustration of biogenic apatite waste utilization to scale up the production of bacterial ALP from the *Bacillus paralicheniformis* strain APSO to the bench-top bioreactor scale for green chemistry sustainability.”

The original Article has been corrected.

